# Morphological Constraints on Double Percolation and Toughening in Nanocarbon-Filled Epoxy–Poly(ether imide) IPN Nanocomposites

**DOI:** 10.1021/acsomega.6c03532

**Published:** 2026-07-26

**Authors:** Matheus Mendes de Oliveira, Rogerio Ramos de Sousa Jr, Daria Strugova, Nicole R. Demarquette, Ragnar Larsson, Roland Kádár, Per Hallander, Danilo Justino Carastan

**Affiliations:** † Center for Engineering, Modeling and Applied Social Sciences, 74362 Universidade Federal do ABC, Santo André 09210-170, Brazil; ‡ Mechanical Engineering Department, École de Technologie Supérieure, Montréal H3C 1K3, Canada; § Industrial and Materials Sciences, 11248 Chalmers University of Technology, Göteborg 412 96, Sweden; ∥ SAAB AB, Linköping 581 88, Sweden

## Abstract

Lightweight polymer nanocomposites with enhanced electrical and mechanical performance are attractive candidates for fuel-efficient aeronautical applications. This study investigates the incorporation and selective localization of graphene nanoplatelets (GNP), carbon nanotubes (CNT), and carbon black (CB) in epoxy and epoxy–poly­(ether imide) (PEI) semi-interpenetrating polymer networks (semi-IPNs). Although the semi-IPN architecture promoted the expected selective localization of the nanofillers within the continuous PEI-rich phase, the anticipated double-percolation effect was not observed. We demonstrate that the main factor driving the loss of nanoparticle connectivity was the presence of epoxy-rich droplets in the PEI-rich phase, which disrupted long-range conductive pathways. The influence of these droplets depended strongly on the nanofiller type, with changes in droplet morphology correlating with increased percolation thresholds and reduced filler connectivity. These results demonstrate that selective localization and phase cocontinuity alone are insufficient to guarantee improved electrical percolation, which requires a favorable mesoscopic morphology. Likewise, impact resistance and fracture mechanisms were also dependent on droplet morphology. A formulation containing 15 PHR of PEI (IPN15) and 2.5 wt % GNP achieved an optimal balance of properties, exhibiting electrical conductivity comparable to that of the conventional epoxy/GNP nanocomposite while providing more than a 250% increase in impact strength. Overall, the findings demonstrate the critical role of mesoscopic morphology in governing the electrical performance of multiphase nanocomposites and highlight both the limitations and potential of epoxy–PEI semi-IPNs as a platform for the development of advanced multifunctional materials.

## Graphical abstract

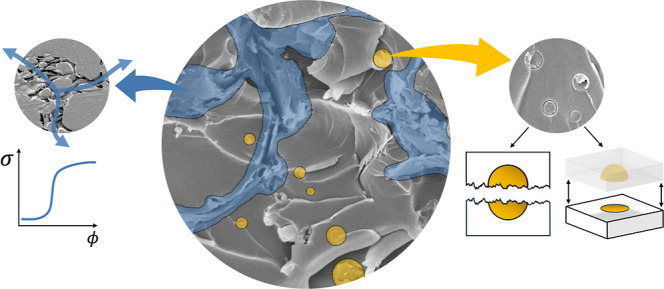

## 1 Introduction

Epoxy resins are valuable for a wide range of applications due to their outstanding properties, including excellent thermal, mechanical, and chemical resistance.^1^ However, the highly cross-linked structure that gives rise to those properties also makes epoxy brittle, leading to poor crack resistance and low fracture toughness at room temperature. Cracks tend to form on the material’s free surfaces and propagate as they absorb energy, eventually leading to fracture.^2,3^ The inherent electrical insulating character of epoxy represents yet another drawback for certain applications. An area where both limitations are particularly critical is in the design of modern airplanes, which are primarily constructed from epoxy-based carbon fiber reinforced polymer (CFRP) composites. These structures must be tough and electrically conductive for electromagnetic shielding and lightning strike protection (LSP), as aircraft are at high risk of being struck by lightning when operating in thunderstorms.^4,5^ Traditional LSP uses metallic meshes that add significant weight (150–200 g m^–2^) and are prone to corrosion, increasing operational costs.^6–8^ Lightweight and electrically conductive nanocomposite materials offer promising novel LSP solutions that not only avoid these costs but also reduce CO_2_ emissions.^9^

Within this context, graphene and other carbon-based nanomaterials, such as graphene nanoplatelets (GNPs), carbon nanotubes (CNTs), and carbon black (CB), have been widely explored as conductive fillers in polymer nanocomposites. In these systems, the electrical percolation threshold (PT) corresponds to the filler content at which electrical conductivity increases abruptly due to the formation of a continuous conductive network. Because of their high intrinsic conductivity and large aspect ratio, graphene and CNTs are among the most effective nanofillers for achieving low PT values. As discussed in a recent review by Lalire et al., the percolation behavior of polymer nanocomposites is strongly influenced by nanofiller dispersion, spatial distribution, and aspect ratio.^10^ For a given nanofiller with a given aspect ratio, several strategies have been proposed to optimize its spatial organization within the matrix, including hybrid filler systems,^11^ surface functionalization^12^ and particle alignment.^13^ Although these approaches differ in implementation, they all aim at modifying the spatial organization and connectivity of the conductive structures, which ultimately governs the formation of conductive pathways. A key observation in this regard is that, while mechanical properties tend to be directly correlated with better dispersion, electrical percolation is known to rely on some level of particle aggregation. Wang et al. recently demonstrated through molecular simulations that excessive graphene dispersion can actually hinder percolation by reducing the number of conductive connections between neighboring particles.^14^ Thus, when designing nanocomposites with low percolation threshold, one must devise strategies that promote a favorable spatial distribution of conductive particles, leading to controlled aggregation and the formation of efficient conductive pathways. For example, Meloni et al. showed that segregated-network architectures in latex lattices can dramatically improve network efficiency and reduce percolation thresholds in reduced graphene oxide (rGO)-filled composites.^15^ Imran et al. also achieved high CNT connectivity by growing them in hollow glass microsphere templates, an arrangement that is particularly effective for electromagnetic interference shielding.^16^ Multiphase polymer systems, particularly immiscible polymer blends, have also been extensively investigated as a means of controlling filler organization through the so-called “double percolation” phenomenon. First reported by Sumita et al. in 1991,^17^ double percolation seeks to reduce the PT by selectively localizing conductive fillers within a minor continuous phase or at the interface between two cocontinuous phases, thereby increasing their local concentration and facilitating network formation. This strategy has been widely and successfully explored.^18–20^

Within this multiphase framework, interpenetrating polymer networks (IPNs) have emerged as a promising platform for polymer-based nanocomposites. IPNs are a class of polymeric systems composed of two or more immiscible polymers with no covalent bonds between their chains, which are at least partially interlocked on a molecular scale.^3^ This system can be achieved by mixing two thermoset polymers (full-IPN) or a thermoset with a thermoplastic polymer (semi-IPN).^21^ Engineering thermoplastics, such as poly­(ether imide) (PEI), are typically used in combination with epoxy resins to form IPNs. The epoxy prepolymer and PEI are initially miscible, then a reaction-induced phase separation (RIPS) occurs after the addition of the hardener, causing the phase morphology to develop until gelation of the epoxy is reached. This results in a multiphase morphology, split between epoxy- and PEI-rich phases, which effectively toughens brittle epoxy resins while maintaining their high thermal resistance and modulus.^22^

In addition to a toughening effect, the biphasic morphology of IPNs can also be exploited to achieve double percolation and reduce PT. Recently, researchers have focused on the incorporation of nanoparticles into thermoplastic-epoxy IPNs as a means to control their morphology and obtain multifunctional materials.^23–25^ However, only a small portion of these studies concerns electrical conductivity and the double percolation phenomenon. Recently, Wang et al. and Liu et al. reported having successfully achieved lower percolation thresholds in epoxy-PEI IPNs reinforced with multiwalled carbon nanotubes (MWCNT)^24^ and silver nanowires (AgNW),^26^ respectively. In both cases, the percolation in the IPN system was significantly reduced when compared to the epoxy-only matrix, and the effect was attributed to a selective localization of the nanoparticles into either the PEI-rich phase (MWCNT) or the epoxy-rich phase (AgNW). It should be noted that the PT reported for MWCNT in epoxy-matrix was 1 wt %, significantly higher than what is typically reported (often less than 0.1 wt %), and the maximum conductivity at 2 wt % was extremely low at only ≃ 10^–9^ S m^–1^, once again out of range for a system that often exceeds >10^–3^ S m^–1^.^27^ Similarly, AgNW-epoxy nanocomposites have been reported with much lower PT (<0.1 wt %) and higher conductivities.^28,29^ This suggests that factors beyond selective localization may have influenced the observed percolation behavior. Ma and colleagues used carbon black (CB) as the nanofiller in an epoxy-PEI semi-IPN and achieved a low PT of 0.5 wt %.^30^ However, the authors did not compare it with an epoxy-only matrix, making it difficult to evaluate the role of double percolation effect, if any. Finally, to the best of the authors’ knowledge, there is a single published study that employed graphene nanoplatelets (GNP) as a conductive filler in the epoxy-PEI system.^31^ In their short paper, Pan et al. report a PT between 0.75 and 1.0 wt %. However, lower PT are commonly reported for conventional GNP-epoxy systems.^10^ The authors also did not compare this result to an epoxy- or PEI-only matrix, and the study lacks a deeper investigation of the material’s morphology.

This study systematically evaluates the feasibility of using the double percolation phenomenon as a strategy to reduce electrical percolation threshold (PT) in nanoreinforced epoxy-PEI semi-IPNs, while simultaneously enhancing toughness compared to conventional epoxy-based nanocomposites. The morphology of the semi-IPN system is thoroughly investigated, both with and without carbon nanofillers (GNP, CNT, and CB). The results reveal that a significant toughening effect can be achieved for certain semi-IPN formulations, but the electrical PT was actually increased by the epoxy-PEI morphology because of a worse connected nanofiller network. Although the study emphasizes GNP-filled composites, detrimental effects on electrical performance are also observed with MWCNTs and CB. Finally, an optimized compromise is identified in which the semi-IPN achieves electrical conductivity comparable to that of the epoxy matrix, along with a significant increase in impact strength. These findings highlight both the limitations and potential of epoxy–PEI semi-IPNs as a platform for the development of multifunctional conductive nanocomposites.

## 2 Experimental Section

### 2.1 Materials

Bisphenol A diglycidyl ether (BADGE) epoxy resin and methyltetrahydrophthalic anhydride (MTHPA) hardener with dimethylbenzylamine (BDA) accelerator were purchased from Avipol (São Paulo, Brazil). FTIR spectra confirmed that no additives or diluents were present (Figure S1). PEI (Ultem 1000) was provided by SABIC’s Innovative Plastics. Graphene nanoplatelets (GNP) were provided by 2Dfab (Sundsvall, Sweden) and used as received. The flakes have an average thickness of 5.64 ± 0.77 nm, as measured by atomic force microscopy (AFM) (Bruker Dimension Icon, Massachusetts, USA), and the average lateral size is 1.68 ± 0.78 μm, as measured by scanning electron microscopy (SEM) (JEOL JCM-6000 Plus, Tokyo, Japan). Raman spectroscopy shows that *I*_D_/*I*_G_ ratio of representative flakes is small (<0.2), which suggests a highly ordered carbon crystalline structure and thus high intrinsic conductivity (Figure S3). Multiwalled carbon nanotubes NC7000 were purchased from Nanocyl, Belgium, and used as received. The average diameter of the tubes was measured by SEM as 12.94 ± 2.47 nm (Figure S4). Conductive carbon black (Vulcan XCmax 22) was purchased from Cabot (USA). Dichloromethane (DCM) was purchased from Vetec (Rio de Janeiro, Brazil).

### 2.2 Sample Preparation

The nanofillers were manually mixed with epoxy resin at different weight contents. Then, the epoxy-nanofiller suspensions were dispersed using different procedures, depending on the nanoparticle: (i) for GNPs, samples were processed for 2 h in an Elmasonics heated ultrasonic bath (Germany) at 60 °C and 37 kHz; (ii) for CNTs, DCM was added and the suspension was processed in a 750 W ultrasonic probe VCX750 from Sonics Vibra-Cell (Newton, USA), using 25% amplitude at the fixed frequency of 20 kHz for 15 min before adding the epoxy resin; and (iii) for CB, the epoxy-CB suspensions were sonicated for 2 h in the ultrasonic bath and then further processed in a Torrey Mills TM65B three-roll mill (3RM) with gaps of 50 μm for 15 min. Different dispersion methods were intentionally employed for each nanofiller in order to optimize their individual dispersion states, rather than applying a single standardized process that might disadvantage certain nanoparticle geometries. Dispersion methods (i) and (ii) were chosen with the goal of optimizing electrical conductivity based on our previous studies,^32^ and 3RM was used in method (iii) due to the high viscosity of CB-epoxy suspensions with high weight contents. For the semi-IPN samples, PEI pellets were dissolved in DCM and added to the epoxy-nanofiller suspensions under magnetic stirring for 10 min. Then, the temperature was raised to 70 °C to evaporate the solvent until all visible evaporation had vanished, and the resulting mixtures were put in a vacuum oven at 100 °C overnight to further remove any remaining solvent. The effectiveness of the solvent removal procedure was assessed by FTIR analysis, which showed no presence of residual DCM in the processed samples, as shown in Figure S2. For both nanocomposites with epoxy-only and IPN matrices, MTPHA hardener was added at 85 PHR (parts per hundred resin) to the mixture under manual stirring at 70 °C for 10 min and then poured into silicone molds. The curing cycle consisted of 2 h at 110 °C and a postcuring step at 130 °C for 12 h. This procedure is summarized in Figure 1. Epoxy-PEI semi-IPNs with a specific PEI content are abbreviated as “IPNXX”, where “XX” represents the PEI content in PHR. When a nanocomposite is prepared with an epoxy-only matrix, the prefix “EP” is used.

**Figure 1:**
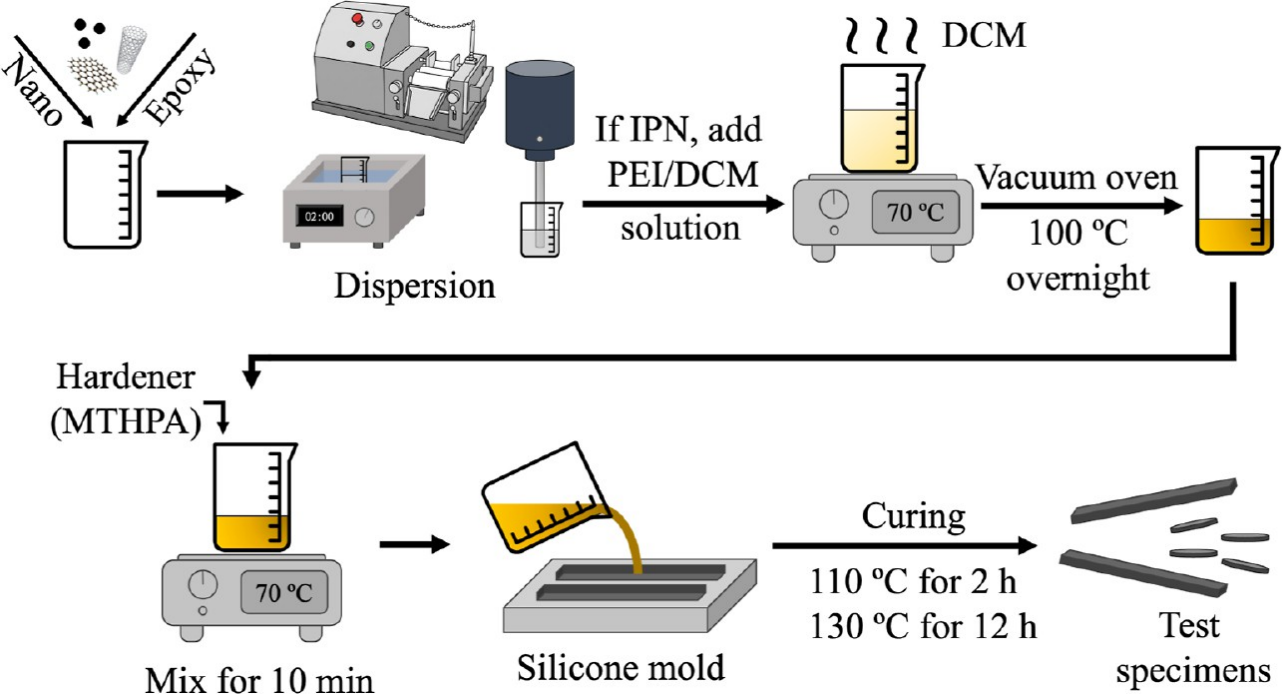
Schematic diagram summarizing the sample preparation process used to produce the nanocomposites.

### 2.3 Characterization

The phase-morphology evolution of semi-IPNs was visually monitored using optical microscopy (OM). Immediately after mixing the components, a drop of material was squeezed between a glass slide and a cover glass and placed in a Linkam T95 heating stage (Salfords, UK) at 110 °C. Images were then taken using an Axio A1 microscope (Carl Zeiss, Oberkochen, Germany).

For cross-sectional images of bulk cured samples, specimens were cut and polished with a suspension of 0.1 g·L^–1^ of fumed silica (Aerosil OX50, Evonik, Germany) in deionized water. The surfaces were then observed in an Olympus OLS4100 confocal microscope (Shinjuku, Japan). For SEM imaging, polished surfaces had the PEI-rich phase etched by lightly rubbing a cotton swab soaked with DCM and then sputter-coated with 15 nm of gold. The images were taken with a FEI Quanta 250 SEM (Thermo Fisher Scientific, Waltham, USA).

Small-amplitude oscillatory shear (SAOS) time sweeps were performed at 110 °C in an MCR 502 rheometer from Anton Paar (Graz, Austria) during the curing reaction, using a constant strain amplitude of 1% and a constant frequency of 1 Hz. Twenty-five mm parallel plates were used with a gap of 0.5 mm.

Dynamic mechanical analysis (DMA) was also performed in the MCR 502 rheometer by sweeping temperatures from 35 to 200 °C at a fixed frequency of 1 Hz and 0.05% deformation, using a torsion fixture. Results were averaged from two test specimens with dimensions of approximately 12.7 × 3.2 × 35 mm.

Electrical conductivity was measured with an SI 1260A gain phase analyzer, coupled with a 1296A dielectric interface, both from Solartron (Leicester, UK). In addition to enabling conductivity estimation over a very broad range, AC dielectric spectroscopy also provides valuable information regarding the underlying conduction mechanisms as a function of filler loading. Dielectric spectra were taken from 0.1 Hz to 1 MHz with an applied AC voltage between 0.5 and 3 *V*_RMS_, depending on the resistivity of the sample. Specimens were 1 mm thick discs with 16 mm in diameter and coated with 20 nm gold on both faces to minimize contact resistance and electrode polarization. The AC conductivity was then calculated from the imaginary permittivity at the lowest available frequency (0.1 Hz) to approximate DC conductivity, using eq 1

1 $$\sigma_{AC}=\omega \varepsilon_{0}\varepsilon^{\prime \prime }\left(\omega \right)$$

where ω is the angular frequency, ε_0_ is the vacuum permittivity, and ε″(ω) is the imaginary permittivity at the applied angular frequency.^33,34^ The electric percolation threshold (ϕ_c_) was obtained by fitting the conductivity data to the linearized power-law relation $\sigma \propto {\left(\phi -\phi_{c}\right)}^{t}$ , where σ is the electrical conductivity, ϕ is the filler content above percolation and *t* is the critical exponent. The resulting fitting parameters are presented together with the percolation data.

Toughness of cured samples was assessed by performing Izod impact testing with unnotched specimens, following the ASTM D4812-19 standard. Specimens were 12.7 × 3 × 63.5 mm and tested in a Tinius Olsen Impact 104 machine (Horsham, USA). Results were averaged from a minimum of five specimens.

## 3 Results and Discussion

### 3.1 Morphology of the EP-PEI Semi-IPNs

Before introducing the nanofillers, the impact of PEI content on the morphology of unfilled semi-IPNs was explored. This is crucial to determine the range where a cocontinuous morphology emerges. Cocontinuous structures have been shown to improve toughness in IPNs^35,36^ and are necessary to achieve the double percolation phenomenon.^34^ For that, samples with different PEI contents were prepared and observed under the optical microscope. The micrographs in Figure 2 show the final morphologies obtained for the different PEI contents tested. It is found that up to 19 PHR the PEI-rich domains (darker gray) form a dispersed phase within a continuous EP-rich phase. In the 20–21 PHR range, the PEI-rich phase forms elongated domains characteristic of cocontinuous morphologies, which is the initial region of interest for this study. At 22 PHR and above, a phase-inverted morphology appears, with EP-rich domains dispersed within a continuous PEI-rich phase.

**Figure 2:**
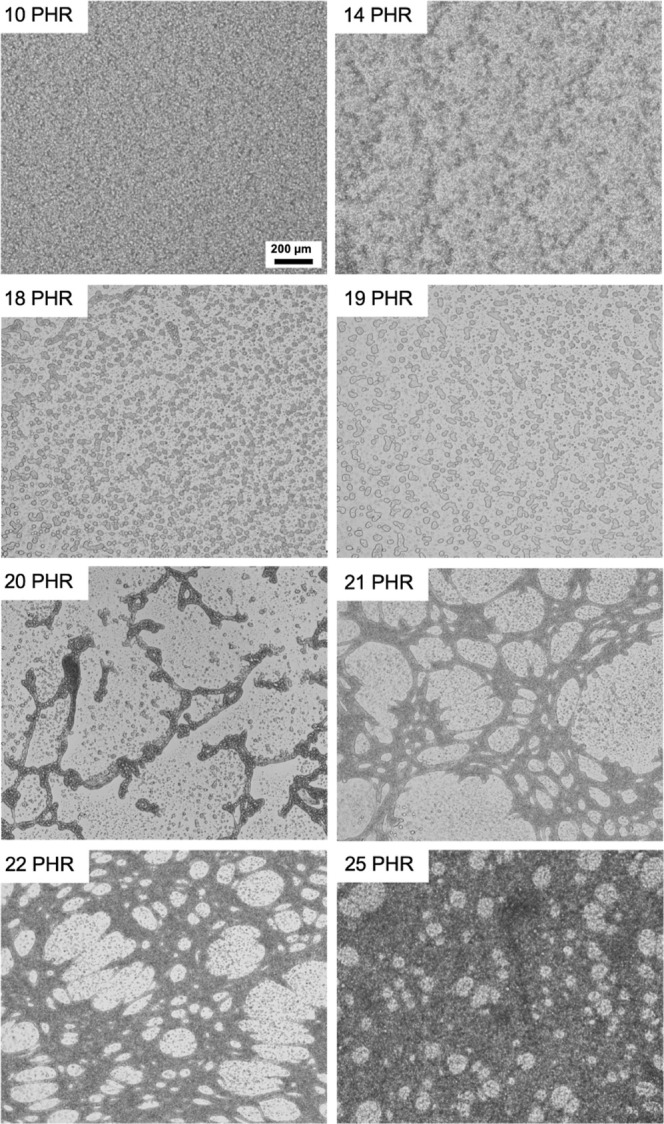
Semi-IPN morphology as a function of PEI content. Dark and light areas are PEI- and epoxy-rich regions, respectively.

Rheology is a powerful tool to monitor phase evolution during the reaction induced phase separation (RIPS). SAOS time-sweeps of complex viscosity (η*) during RIPS are shown in Figure 3a. At 0 PHR of PEI (neat DGEBA-MTHPA), the curing behavior is typical of epoxy resins: a slow increase in viscosity during the induction phase (up to 15 min), followed by a sharp rise from 15 to 35 min due to cross-linking, and then it saturates due to gelation, halting monomer mobility.^37^ At 10 PHR, a viscosity increase appears around 12 min (prior to cross-linking), indicating spinodal decomposition rather than nucleation and growth,^38^ consistent with OM micrographs taken during RIPS for other PEI contents (Figure S5). As the PEI content increases, this viscosity peak increases and broadens, eventually merging with the cross-linking rise to form a shoulder. Higher PEI contents accelerate phase separation because the solubility limit is reached earlier. Rheology detects this onset more sensitively than OM, highlighting earlier phase separation at higher PEI levels. Together, rheology and OM offer complementary insights into morphology and RIPS kinetics.

**Figure 3:**
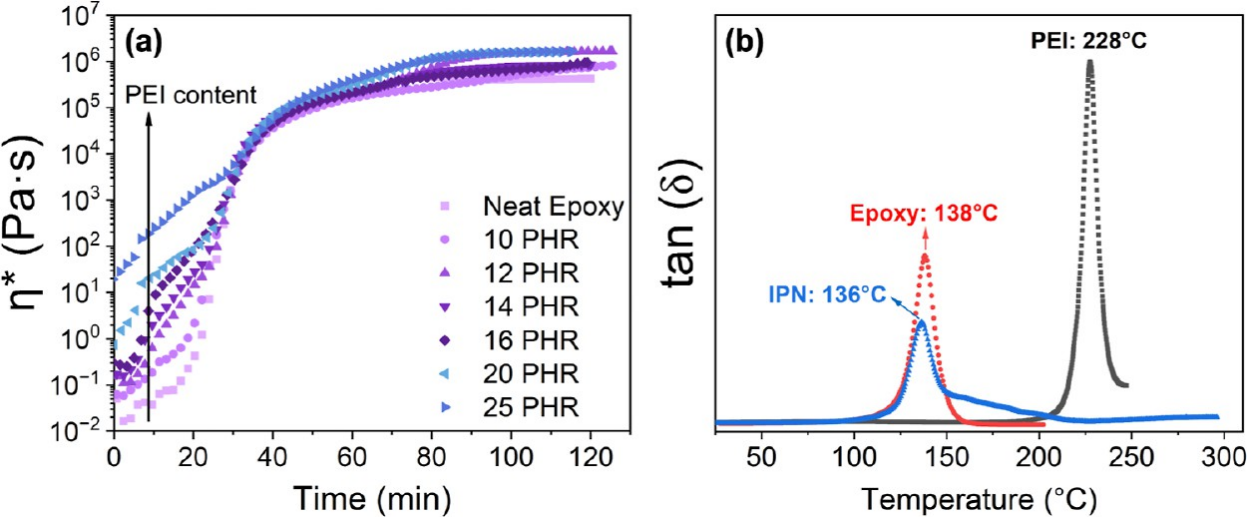
(a) Complex viscosity evolution during RIPS of EP-PEI semi-IPNs prepared with various contents of PEI; (b) DMA curves of the loss factor tan­(δ) as a function of temperature for pure epoxy (DGEBA-MTHPA), PEI and a cocontinuous semi-IPN with 20 PHR of PEI.

At this stage, DMA was used to further assess the molecular-level entanglement between epoxy and PEI chains in the cured material with a cocontinuous morphology. Figure 3b presents tan­(δ) measured during temperature sweeps for neat epoxy, neat PEI, and for the cocontinuous 20 PHR semi-IPN. The *T*_g_ can be estimated as the maximum temperature in tan­(δ),^39^ where the *T*_g_ of the cross-linked epoxy was found to be 138 °C, while the PEI’s *T*_g_ is significantly higher, at 228 °C. As expected, the semi-IPN displays two relaxation phenomena, with a tan­(δ) peak at 136 °C and a wide and diffuse shoulder that stretches from around 150 to 200 °C. This broadened, lower and shifted tan­(δ), combined with the absence of a peak at the original PEI’s *T*_g_, suggests that virtually all PEI chains are closely mixed with epoxy.^40–42^ The PEI-rich regions are mostly composed of nanodomains with a wide range of EP-PEI compositions. At the same time, epoxy’s tan­(δ) remains virtually unchanged, indicating that the epoxy-rich domains are almost entirely composed of epoxy.

### 3.2 Nanofiller Selective Localization

A PEI content of 20 PHR was chosen to proceed with the initial tests involving the carbon nanofillers due to its cocontinuous morphology. The wetting coefficient (ω_a_) has been widely used in the polymer blend field to predict which phase the filler would migrate to, and it has also been applied for semi-IPN systems.^30,43,44^ It can be calculated using eq 2

2 $$\omega_{a}=\frac{\gamma_{F-B}-\gamma_{F-A}}{\gamma_{A-B}}$$

where γ_F–A_, γ_F–B_ and γ_A–B_ are the interfacial tensions between the filler and polymer A, between the filler and polymer B, and between polymer A and polymer B, respectively. The surface energy for each component is shown in Table 1, from which the interfacial tension between the different pairs (γ_pair_) was calculated using the harmonic mean average

3 $$\gamma_{12}=\gamma_{1}+\gamma_{2}-4\left(\frac{\gamma_{1}^{d}\gamma_{2}^{d}}{\gamma_{1}^{d}+\gamma_{2}^{d}}+\frac{\gamma_{1}^{p}\gamma_{2}^{p}}{\gamma_{1}^{p}+\gamma_{2}^{p}}\right)$$

where γ_*i*_ is the surface energy of component *i*, γ_*i*_^*d*^ and γ_*i*_^*p*^ are the dispersive and polar parts of the surface energy of component *i*, respectively, and γ_*i*_ = γ_*i*_^*d*^ + γ_*i*_^*p*^.^24,45,46^ The calculated ω_a_ for each system is shown in Table 2. The wetting coefficient predicts three possibilities: if ω_a_ > 1, the filler particles will preferentially be located in polymer A; if ω_a_ < −1, they would prefer polymer B instead; and if −1 < ω_a_ < 1 the filler will preferentially be distributed along the interface between the phases.^17^ Since we have ω_a_ > 1 for all nanocomposites, the PEI-rich phase is predicted to be the one toward which the nanoparticles should migrate during the RIPS process. It must be noted that the implementation of this approach in the present context is highly simplified, as it relies on equilibrium conditions and literature values that do not account for surface functionalization, impurities, or processing-induced changes in the nanofillers. However, its purpose is only to provide a first-order thermodynamic approximation of the preferential migration tendency of the nanofillers during phase separation. Also, the wetting coefficient analysis is based on equilibrium thermodynamic considerations, whereas the present system evolves under RIPS, in which viscosity continuously increases during curing. As polymerization progresses, the increasing viscosity and network formation progressively restrict nanofiller mobility, limiting their ability to migrate toward the thermodynamically preferred phase. Therefore, the final nanoparticle localization is determined not only by thermodynamic affinity, but also by kinetic effects associated with diffusion limitations and phase separation dynamics.

**Table 1:** Surface Energy (γ) and Its Dispersive and Polar Parts (γ_*i*_^*d*^ and γ_*i*_^*p*^, Respectively) for Each Component of the Nanofilled Semi-IPN Samples

component	γ (mN/m)	γ^*d*^ (mN/m)	γ^*p*^ (mN/m)
PEI^24^	43.5	37.7	5.8
DGEBA^24^	52.0	47.3	4.7
MWCNT^47^	28.0	23.1	4.9
CB^30^	34.4	28.8	5.6
GNP^48^	54.8	41.6	13.2

**Table 2:** Interfacial Tension Between Each Possible Pair (γ_pair_) and the Calculated Wetting Coefficient (ω_a_) for Each Nanocomposite

nanofiller	possible pairs	γ_pair_ (mN/m)	ω_a_
CB	CB-PEI	1.19	2.82
	CB-DGEBA	4.58	
	PEI-DGEBA	1.20	
MWCNT	MWCNT-PEI	3.58	3.95
	MWCNT-DGEBA	8.32	
	PEI-DGEBA	1.20	
GNP	GNP-PEI	3.07	1.11
	GNP-DGEBA	4.40	
	PEI-DGEBA	1.20	

The thermodynamic theoretical predictions were confirmed experimentally. Figure 4 showcases how the GNP flakes remain in the PEI-rich phase during the RIPS process, staying out of the surrounding epoxy-rich regions that are precipitating and coalescing nearby. Over time, as the PEI-rich phase becomes more refined, the GNP flakes naturally move closer together. Figure 5 shows OM images in which the selective localization of GNP, CNT and CB exclusively into the PEI-rich phase (darker regions) can be seen throughout the imaged area.

**Figure 4:**
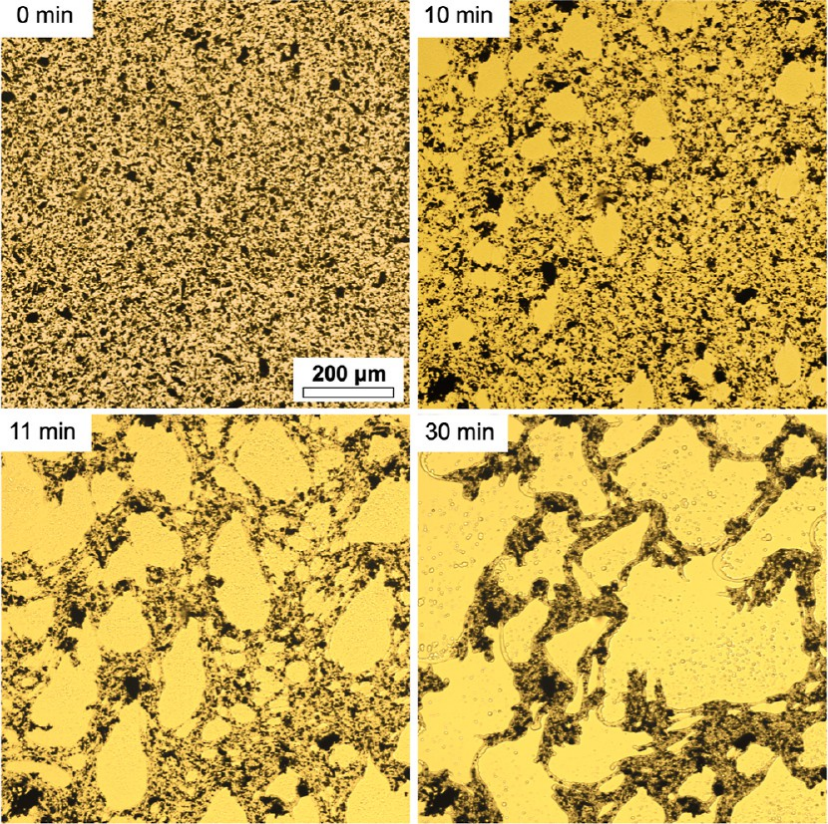
Optical micrographs of IPN20 GNP 1.0 wt % taken at different times during the RIPS process.

**Figure 5:**
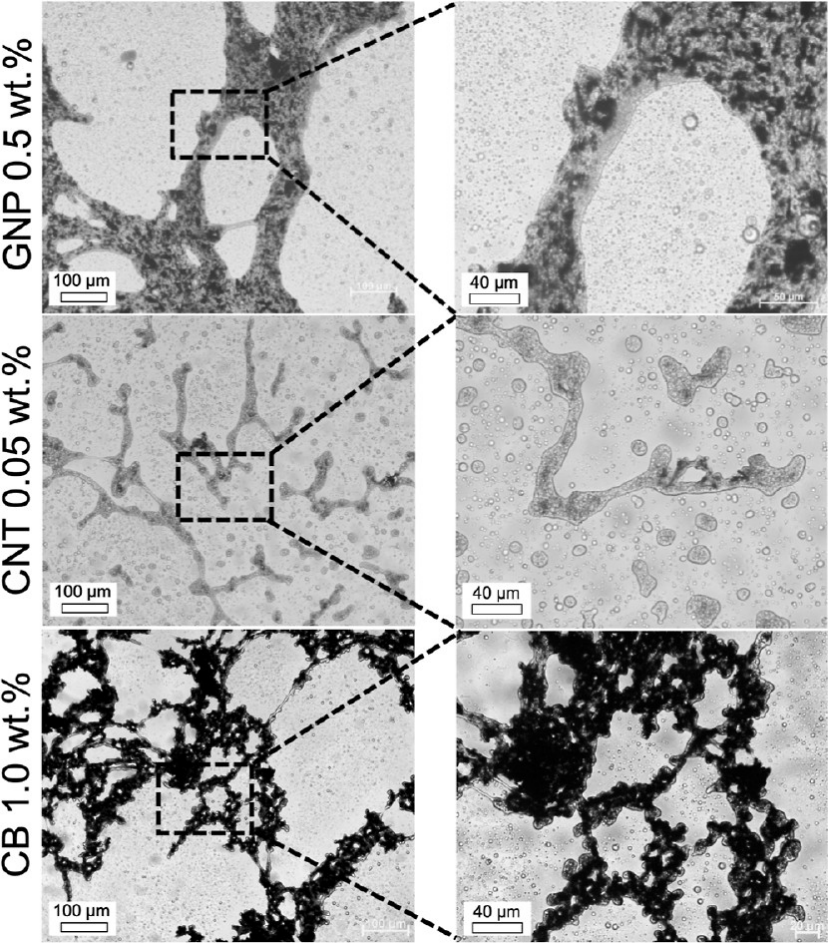
Optical micrographs of the final morphologies achieved after RIPS of IPN20 samples containing different carbon nanofillers. Higher magnification on the right highlights their selective localization in the PEI-rich phase (darker gray).

DMA was used to confirm the localization of GNP in the bulk by analyzing its effect on the phase-specific relaxation behavior. Epoxy matrices and semi-IPNs containing varying GNP loadings were compared. Figure 6 shows temperature sweeps of the shear loss modulus G″, the peak of which can also be used to determine the material’s *T*_g_.^39^ Insets show how *T*_g_ of epoxy (a) and epoxy-rich phase (b) varies with GNP content. In neat epoxy, the single relaxation peak shifts to higher temperatures as the GNP content increases to 1.0 wt %, then drops at higher loadings. This is common in GNP-epoxy systems, due to an initial mobility restriction at lower loadings followed by interfacial free volume caused by agglomerations at higher contents.^49^ In semi-IPNs, however, G″ curves show a stable epoxy-rich peak and a shifting PEI-rich relaxation. The epoxy-rich peak barely moves (<2 °C), while the PEI shoulder shifts significantly and increases in magnitude, indicating that the GNPs are stiffening the PEI-rich phase almost exclusively. This phase-selective stiffening behavior is useful for assessing phase-separated nanocomposites at a more representative scale and, to the best of our knowledge, has not been previously reported.

**Figure 6:**
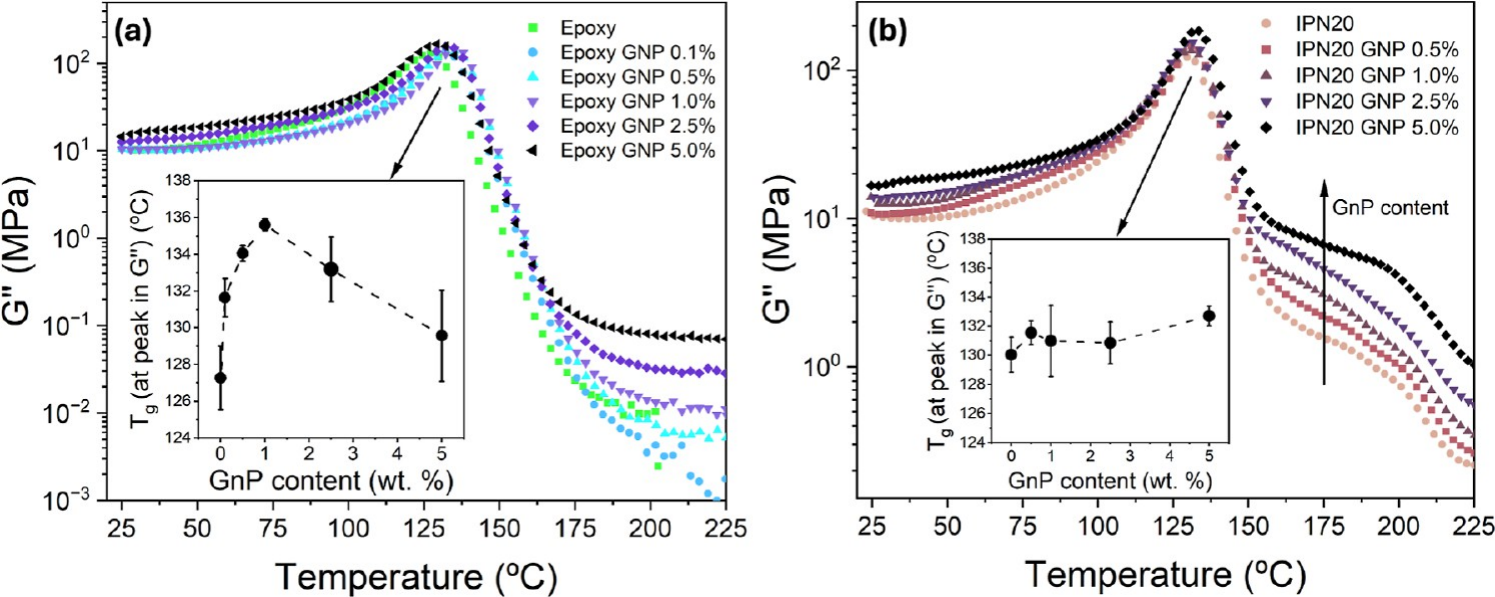
G″ as a function of temperature for epoxy-GNP (a) and IPN20-GNP (b) nanocomposites at different GNP loadings. Insets show how *T*_g_ of epoxy (a) and epoxy-rich phase (b) varies with GNP content.

### 3.3 Morphology of Nanocarbon-Filled Semi-IPNs

The morphology in fully cured, bulk semi-IPN samples was assessed by confocal microscopy and SEM. After cutting and polishing the surface of the cross sections, the confocal micrographs displayed on the left column in Figure 7 were taken. A similar cocontinuous morphology that was observed in OM is also observed, where elongated PEI-rich phases (light gray) are dispersed in a continuous EP-rich phase. With the higher resolution of the confocal laser microscope, small epoxy-rich droplets dispersed within the PEI-rich phase can be seen. This is due to a secondary phase-separation process, likely due to the entrapment of epoxy monomers and oligomers within the PEI-rich region that was vitrified after a certain reaction extent was reached, and the same happened with PEI molecules that were trapped in the epoxy-rich phase.^36^ All published works reporting images of the DGEBA-PEI morphology on this length scale have also found these features.^24,26,36^ The impact of each nanofiller on the semi-IPN morphology will be discussed separately below.

**Figure 7:**
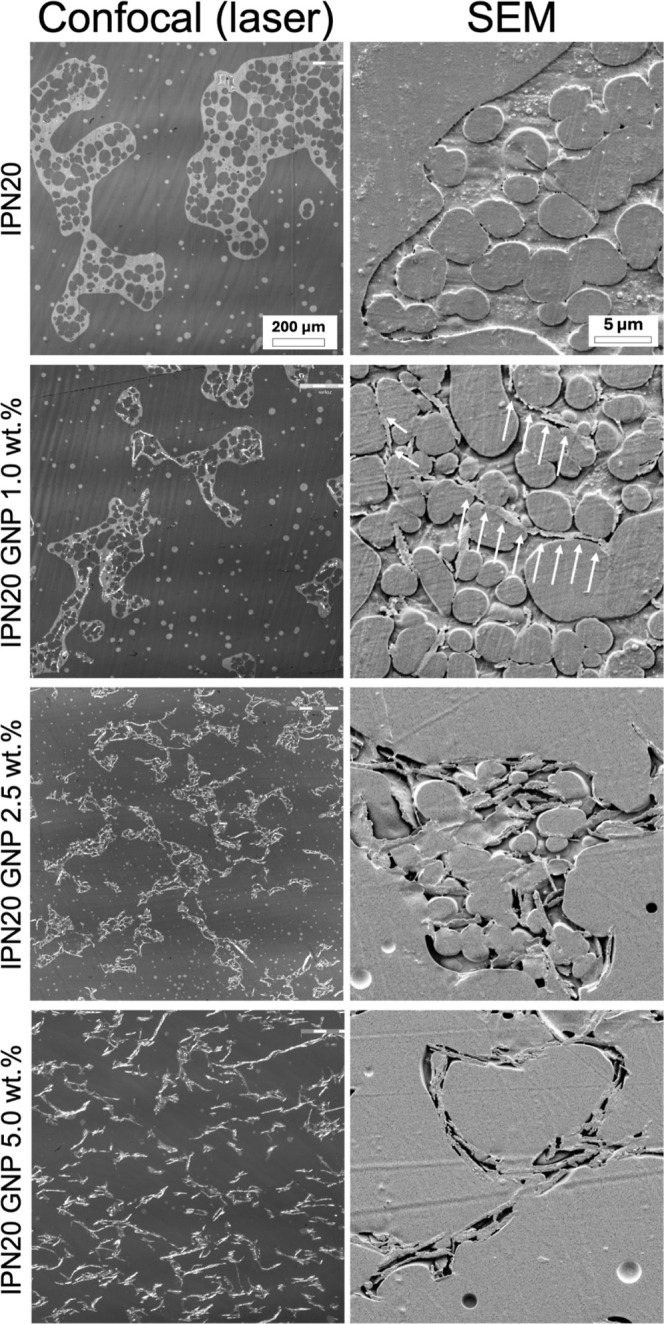
Confocal laser micrographs of GNP-filled semi-IPNs at different weight fractions (left) and SEM micrographs at higher magnification of the same specimens (right). Arrows are highlighting the position of GNP flakes that assemble into short paths.

#### 3.3.1 GNP-Filled Semi-IPNs

The larger GNP flakes are clearly seen in the confocal micrographs as bright lines occupying exclusively the PEI-rich phase, squeezed between the outer continuous epoxy-rich region and the epoxy-rich droplets. With increasing GNP content, the bicontinuous morphology becomes progressively more refined, and at 5 wt % of GNP the PEI-rich phase around the flakes is so thin that it is difficult to visualize. The refining effect that rigid nanofillers cause in conventional polymer blends is well documented and is usually attributed to the formation of a rigid layer of particles at the interface that prevents or hinders the coalescence of that phase.^50^ However, other mechanisms may occur with the addition of nanoparticles that also lead to morphology refinement, including: reduction of interfacial tension, modification of the viscosity ratio between the blend components (due to the preferential location of the particles), formation of interconnected nanoparticle networks in the matrix that reduce the mobility of the dispersed phase droplets, and steric hindrance.^51^ Additionally, there is a significant reduction in the number and size of the epoxy-rich droplets with GNP addition. At 5 wt %, the total surface area of the flakes is large enough that the PEI-rich phase that wets them spreads out and forms only a thin layer. This might prevent secondary phase separation by allowing excess epoxy monomers to still migrate back into the epoxy-rich phase, rather than being trapped in the PEI-rich phase and then precipitating as droplets later on in the RIPS process. As a result, almost no epoxy-rich droplets are observed in this sample.

The same samples had the PEI-rich phase etched on the surface and were observed under SEM to produce the micrographs shown on the right column in Figure 7. Although some flakes were likely removed with the PEI etching, the higher magnification allowed for visualization of the flakes’ arrangement and how connected or not they are. Up to 1.0 wt % loading, the morphology of the PEI-rich phase does not significantly differ from that of the nonreinforced semi-IPN. The flakes occupy the small spaces between the droplets, where two competing effects seem to occur: on the one hand, they are pushed against each other and are seen to form well-defined end-to-end chains that would be beneficial to electrical conduction (as highlighted by white arrows in Figure 7); on the other hand, these chains are abruptly cut short by droplets that stand in their way and are prevented from forming long-range networks. At 2.5 wt %, the increase in the number of flakes and the reduction in droplets result in a different arrangement. The flakes are still squeezed together, but now they are in closer contact throughout the PEI-rich phase and form continuous networks. Finally, at 5.0 wt %, the GNP flakes form tightly packed networks, wrapped by a thin PEI-rich phase, with virtually no droplets present.

#### 3.3.2 CNT-Filled Semi-IPNs

SEM micrographs of CNT-filled nanocomposites are displayed in Figure 8. Comparing the micrograph of the sample with the lowest CNT content tested (a) (0.01 wt %) to the nonreinforced 20 PHR semi-IPN (seen in Figure 7) it is clear that the epoxy-rich droplets are more fused together, rather than existing as distinct spheres. Polymerization kinetics have a direct influence on the morphology of the phase separation in IPN systems. Recent studies have shown that factors such as phase mobility before and after gelation can affect droplet coarsening, coalescence, and connectivity, and even trigger transitions between spinodal decomposition and nucleation-and-growth mechanisms.^52^ Therefore, the presence of CNTs likely altered the kinetics of phase separation and morphology evolution, but the exact mechanisms involved are beyond the scope of this work. At this low concentration, CNTs were well dispersed without forming large bundles, making individual CNTs difficult to spot, but the morphology seems to be hindering long-range connection by creating pockets of isolated PEI-rich regions. At CNT concentrations above electrical percolation, such as 0.15 wt % displayed in Figure 8b, the increase in viscosity of the PEI-rich phase leads to fewer and more spaced-out droplets, allowing for more CNT-to-CNT connection, but the droplets are still prevalent in the morphology. Due to the higher filler concentration, bundles of CNTs are now visible within the PEI-rich pockets between the epoxy-rich droplets, as indicated by white arrows.

**Figure 8:**
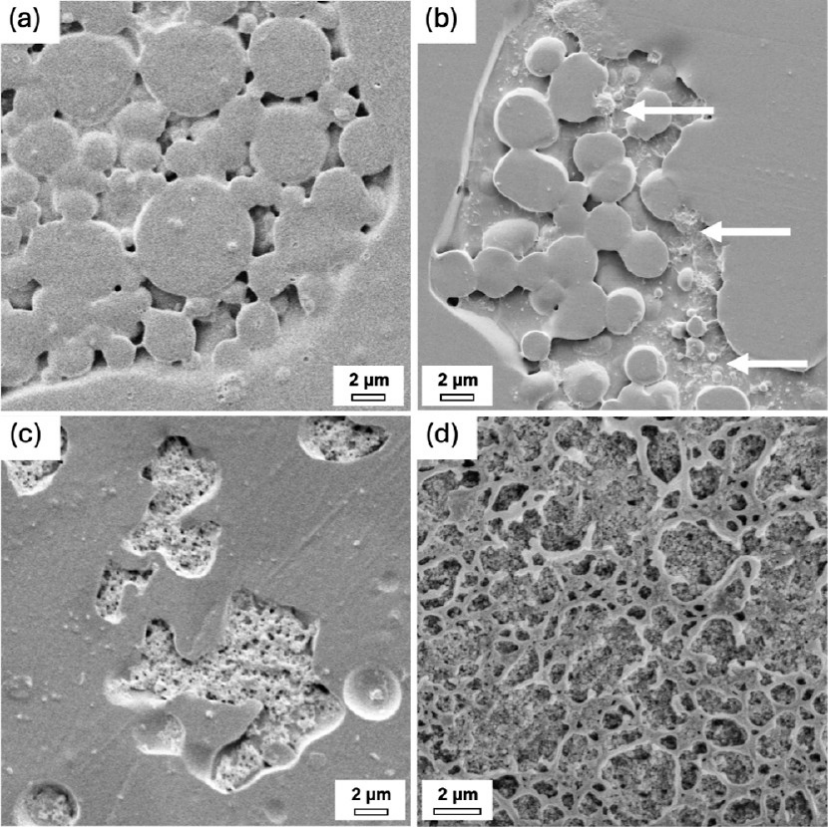
SEM micrographs of CNT and CB-filled IPN20 nanocomposites at two loadings, before and after electrical percolation. (a,b) Show CNT-filled samples at 0.01 and 0.15 wt %, respectively, while (c,d) display CB-filled samples at 1.0 and 10 wt %, respectively. White arrows point to CNT bundles.

#### 3.3.3 CB-Filled Semi-IPNs

The addition of 1 wt % of CB particles caused a noticeable change in the semi-IPN’s morphology (Figure 8c). Although elongated PEI-rich phases are still present, they are smaller and more spaced out. Interestingly, the epoxy-rich droplets resulting from secondary phase separation—seen in the GNP- and CNT-filled samples—are virtually absent. This result contrasts with what was observed in the only published paper that also employed CB as a nanofiller in EP-PEI semi-IPNs, where epoxy-rich droplets are still visible at the same concentration of 1 wt % of CB.^30^ Although the filler used was also an amorphous CB from Cabot, the grade is different; thus, the surface energy and/or surface area might also differ. As discussed previously, in the case of GNP-filled samples, the suppression of secondary phase separation with increasing GNP weight content appears to be caused by an increase in the total surface area of the nanofiller. Thus, the highly porous structure of the CB aggregates stretches the PEI-rich layer around them and allows epoxy monomers to escape. At 10 wt % loading, a cocontinuous morphology structure is seen, in which the PEI-rich phase loaded with CB percolates through the sample. The PEI etching reveals CB particles that form a closely connected network in Figure 8d.

### 3.4 Electrical Percolation

#### 3.4.1 GNP-Filled Nanocomposites

Electrical percolation curves were obtained for nanocomposites based on epoxy and IPN20 matrices and subsequently compared. The conductivity curves of GNP-filled nanocomposites, shown in Figure 9a, display the typical percolation behavior described by the power-law relationship $\sigma \propto {\left(\phi -\phi_{c}\right)}^{t}$ . Below ϕ_c_, the insulating polymer matrix dominates the electrical response, resulting in little change in conductivity with increasing filler content. Once ϕ_c_ is exceeded, conductivity increases nonlinearly by several orders of magnitude as an interconnected network forms, eventually reaching a saturation plateau. By fitting the power-law, ϕ_c_ for the GNP-filled epoxy was determined to be 0.48 ± 0.007 wt %. This value lies on the lower end of those reported for most GNP/epoxy nanocomposites, which typically percolate at GNP contents exceeding 1 wt %.^53^ The low ϕ_c_ obtained here suggests a favorable combination of nanofiller quality, dispersion, and distribution within the epoxy matrix. The σ_AC_ spectra in Figure 9b further highlight the insulator-to-conductor transition. Below electrical percolation, the conductivity exhibit a strong frequency-dependent behavior. Higher frequencies promote nonohmic conduction mechanisms, such as electron hopping and tunneling between GNP flakes that are separated by a thin, insulating matrix layer. As network connectivity increases, a frequency-independent plateau appears, indicating that neighboring particles are sufficiently close for charge transport to occur predominantly through ohmic conduction.^49,54^ Thus, the σ_AC_ of samples with GNP contents below ϕ_c_ are strongly dependent on frequency, with insulating dielectric behavior. After percolation is achieved at 1.0 wt % loading, a σ_AC_ plateau is seen for most of the frequency range, and at 2.5 wt % it becomes completely frequency-independent, displaying a predominantly ohmic conduction mechanism.

**Figure 9:**
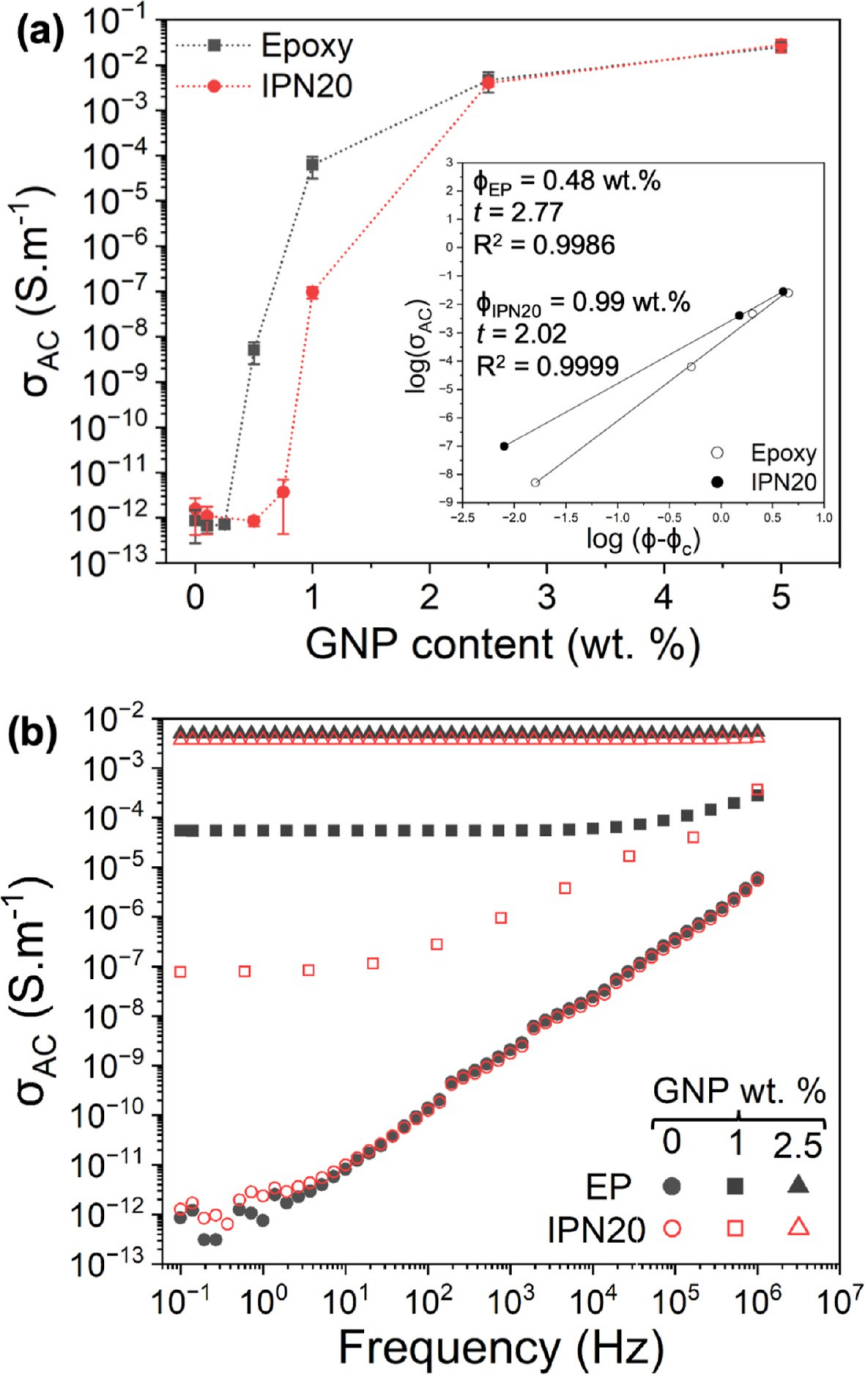
Electrical percolation of GNP in epoxy and IPN20 matrices, along with their respective power-law fitting parameters in the inset (a); σ_AC_ spectra of selected conditions (b).

When IPN20 is used as a matrix, an increase in ϕ_c_ is observed rather than the expected reduction through the double-percolation effect, and ϕ_c_ virtually doubles to 0.99 wt %. Despite this increase, the conductivity achieved after percolation matches the values obtained with the epoxy matrix, saturating at about 3 × 10^–2^ S m^–1^. It is important to note that the additional interfaces introduced by the multiphase system can influence charge transport. As recently discussed by Jagadish et al., interfaces may introduce energy barriers that hinder charge transfer between neighboring conductive domains, giving rise to additional contact and interfacial resistances within the conductive network.^55^ Such effects can reduce the efficiency of charge transport through an established network, even when favorable nanofiller localization is achieved. However, these factors primarily influence charge transport through an already established network.^56^ The fact that both matrices achieve nearly identical saturation conductivities suggests that differences in interfacial resistance between the PEI-rich phase and neat epoxy are not significant in this system. Instead, the results indicate that the intrinsic conductivity of the GNP conductive networks, once established, remains largely unaffected by the surrounding phases.

Thus, to understand the increase in percolation threshold observed in the semi-IPN system, it is important to recall the main factors governing electrical percolation in nanocomposites. As discussed earlier, percolation behavior is primarily influenced by nanofiller dispersion (the extent to which agglomerates are broken down), nanofiller spatial distribution (including particle/aggregate arrangement and distances), and nanofiller aspect ratio.^10^ Among these factors, the aspect ratio can be ruled out, as it remains unchanged between the systems. Likewise, GNP dispersion is not expected to be a major contributor, since the same processing route was used for both matrices and confocal/SEM micrographs do not reveal GNP agglomerates. Therefore, differences in spatial distribution is the most plausible explanation. As alluded to before, the presence of epoxy-rich droplets in the PEI-rich phase appears to disrupt long-range nanoparticle connectivity (Figure 7), hindering the formation of an effective conductive network and consequently increasing the percolation threshold. Analyzing the σ_AC_ spectra in (b) further demonstrates the differences in connectivity between matrices. At 1.0 wt % GNP, while the epoxy system has already achieved a σ_AC_ plateau, the semi-IPN still exhibits strong frequency dependence due to polarization, electron-hopping, and tunneling effects as electrons still need to travel through portions of the insulating matrix. Frequency-independent behavior was only achieved at 2.5 wt % and above, where the semi-IPNs’ spectra matched those of the epoxy-based nanocomposites.

#### 3.4.2 CNT- and CB-Filled Nanocomposites

The CNT and CB-filled nanocomposites also had their percolation behavior compared between epoxy and IPN20 matrices. For both nanoparticles the semi-IPN’s detrimental effect was also present, though in distinct ways.

The CNT-epoxy samples percolated at a much lower weight fraction compared to the GNP-filled nanocomposites, only 0.016 wt %. This behavior is primarily attributed to the extremely high aspect ratio of CNTs. The obtained value lies at the lower end of the range typically reported for CNT/epoxy nanocomposites,^27^ and the saturation conductivity at 0.2 wt % reached about 2 × 10^–1^ S m^–1^, which is also typical. However, for CNT-filled semi-IPNs, electrical percolation could only be achieved at 0.085 wt %, which, despite remaining relatively low, represents a 5-fold increase. Equally important, the saturation conductivity is about 2 × 10^–6^ S m^–1^, a 5 orders of magnitude decrease. As observed in the micrographs in Figure 8, pockets of isolated PEI-rich regions are created by fused epoxy-rich droplets. At high CNT loadings, CNT bundles are seen confined within these pockets. This morphology severely hinders the formation of an interconnected CNT network throughout the semi-IPN matrix, as the conductive pathways become spatially interrupted by the phase-separated structure. As a result, complete electrical percolation is not achieved. This interpretation is further supported by the σ_AC_ spectra shown in Figure 10b. Even at CNT concentrations above ϕ_c_, σ_AC_ still exhibits noticeable frequency dependence instead of a well-defined low-frequency plateau expected for fully percolated systems with dominant ohmic conduction. This behavior indicates that charge transport is still governed to a significant extent by electron hopping and tunneling between disconnected or poorly connected CNT domains, confirming that a continuous conductive network was not fully established in the semi-IPN morphology.

**Figure 10:**
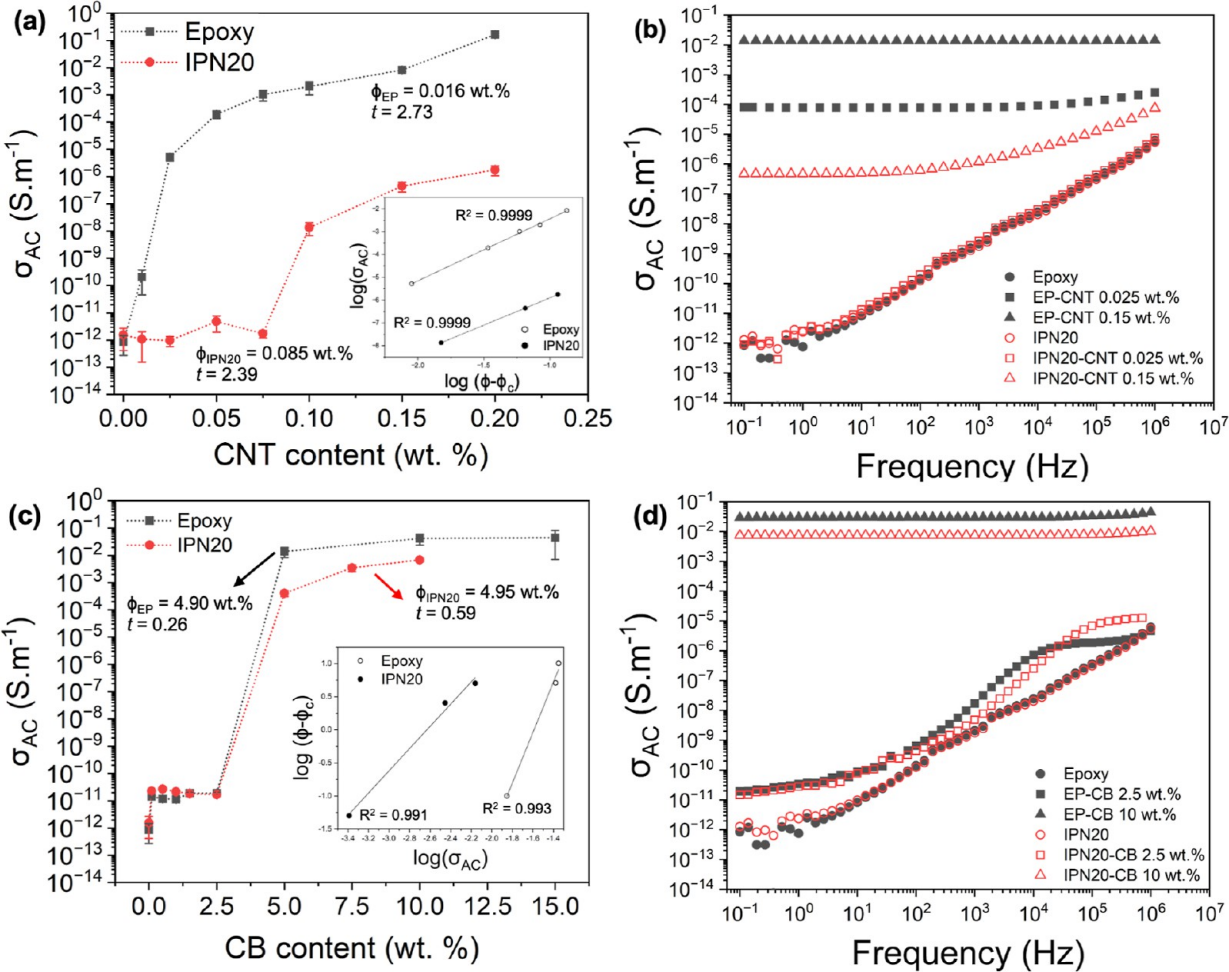
Electrical percolation of CNT- (a) and CB-filled (c) nanocomposites with epoxy and IPN20 matrices, along with their respective power-law fitting parameters. Their σ_AC_ spectra at loadings before and after percolation are displayed in (b) and (d), respectively.

CB-filled nanocomposites exhibit a distinct behavior that further supports the negative impact of epoxy-rich droplets on nanoparticle connectivity of GNP- and CNT-filled samples. As shown in Figure 10c, the calculated ϕ_c_ for the CB-filled samples was 4.90 wt % in epoxy and 4.95 wt % in the semi-IPN matrix. The ϕ_c_ values are relatively low considering that reported percolation thresholds for CB-filled polymer nanocomposites typically span a broad range from approximately 3 to 30 wt %.^57,58^ Most importantly, they are virtually the same in both systems, and a clear deleterious effect on percolation was not observed in this case. In addition, the corresponding σ_AC_ spectra display virtually the same frequency dependence in both matrices, indicating that network formation is essentially unaffected by the matrix morphology. The only significant difference lies in the saturation conductivity, which is approximately 1–2 orders of magnitude higher in the epoxy matrix. This reduction in conductivity in the CB-filled IPN20 nanocomposites may indicate increased contact resistance between neighboring nanoparticles or higher interfacial resistance, which can limit charge transport even when a conductive network is already established.^59^ These results suggest that, in the absence of epoxy-rich droplets, the connectivity of the CB particles is not significantly disrupted. Consequently, the nanoparticles remain free to establish a continuous long-range conductive network, leading to nearly identical percolation behavior in both matrices.

#### 3.4.3 Epoxy-Rich Droplets’ Role in Disturbing Nanoparticle Connectivity

In order to further assess the adverse effect of epoxy-rich droplets on nanofiller connectivity, a quantitative analysis of droplet size and prevalence was conducted using cross-sectional SEM images of the GNP- and CNT-filled IPN20 samples (Figures S8 and S9). Details of the procedure are provided in the Supporting Information. The results are presented in Figure 11, overlaid with the electrical percolation curves.

**Figure 11:**
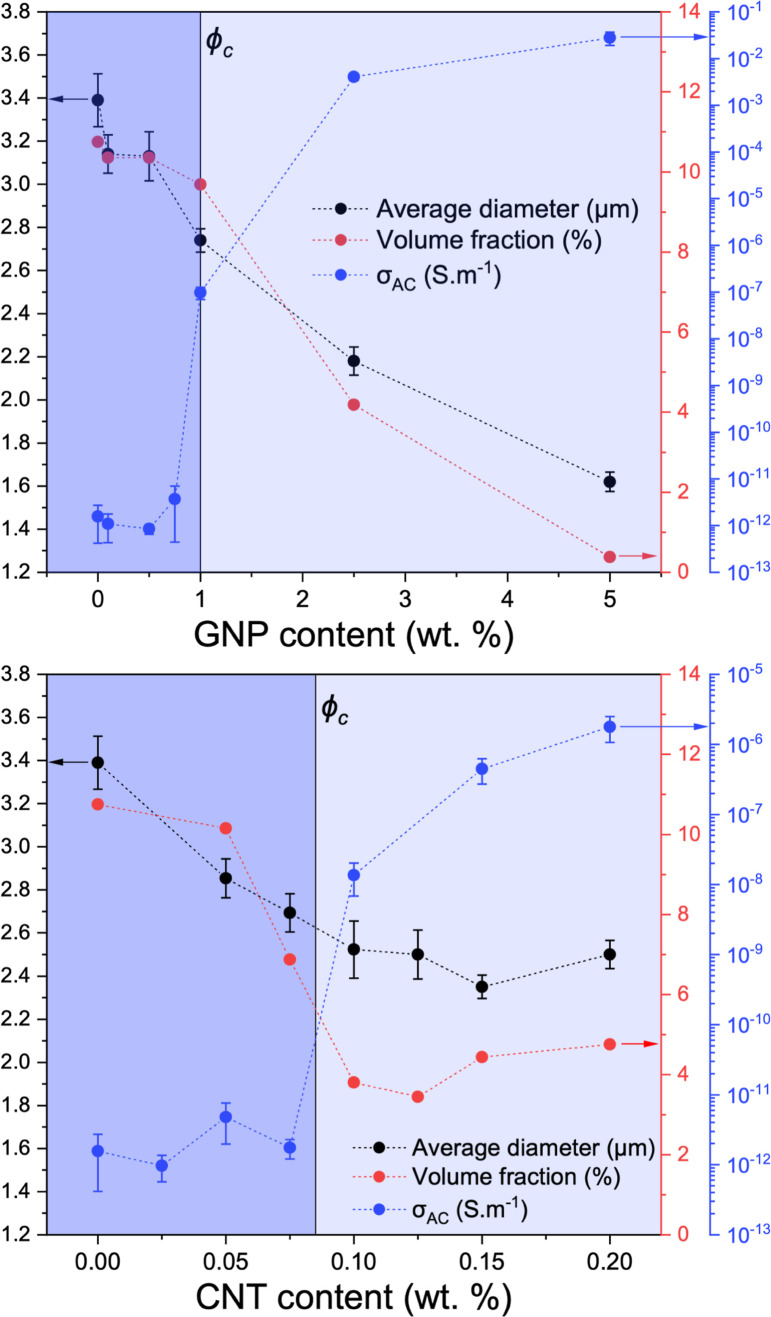
Quantitative analysis of the average diameter and volume fraction of epoxy-rich droplets in GNP- and CNT-filled IPN20 nanocomposites as a function of nanofiller loading, together with their corresponding electrical conductivity and percolation threshold (ϕ_c_).

For the GNP-filled semi-IPNs, both droplet size and volume fraction decrease sharply near the onset of electrical percolation. The average droplet diameter decreases from 3.4 to 1.6 μm and the volume fraction occupied by the epoxy-rich phase drops from 10.8 to 0.4 vol %, virtually vanishing. This substantial reduction leaves more continuous space for GNP flakes to establish conductive pathways. As the droplets are suppressed, connectivity increases and the conductivity ultimately reaches the same level as that of the epoxy-only matrix.

In contrast, the CNT-filled systems exhibit a more gradual reduction in average droplet diameter with increasing CNT loading, eventually stabilizing at approximately 2.5 μm. The droplet volume fraction remains high up to 0.05 wt % CNT, after which it decreases sharply from approximately 10 to 4 vol %, coinciding with the electrical percolation threshold. This simultaneous occurrence strongly suggests a correlation between CNT network formation and the reduction in droplet volume fraction. Despite this decrease, the droplet volume fraction in the percolated CNT-filled samples remains substantially higher than that of the percolated GNP-filled systems (4 vol % versus 0.4 vol %). This difference is consistent with the lower conductivity observed in the CNT-filled nanocomposites after percolation and is further supported by the pronounced frequency dependence of their electrical response, which indicates a poorly connected conductive network.

These results, along with the observation that percolation behavior of CB-filled samples without droplets was not affected, provide further evidence that the presence and persistence of epoxy-rich droplets is the primary factor governing the increase in electrical percolation.

#### 3.4.4 Cocontinuity of the Semi-IPN Morphology

In addition to the epoxy-rich droplets that hinder the nanofiller connection, another factor that could potentially have influenced the electrical performance is the PEI-rich continuity. Previously, cocontinuity in the IPN20 matrix was assessed by cross-sectional microscopy, offering limited two-dimensional insight into a complex 3D structure.

Thus, a solvent extraction method was employed: samples with varying sizes were immersed in DCM for 12 h to selectively dissolve the PEI-rich phase. If the remaining part is self-supporting and its mass matches the nominal phase content, then the material is considered cocontinuous. Otherwise, cocontinuity degree is estimated as the ratio of extracted mass to expected content.^60^ The tests in unfilled IPN20 and GNP-filled samples below percolation (0.10–0.50 wt %) showed a consistent mass loss of around 40%, despite the nominal PEI content being only ∼9.7% (Table S1). This higher loss is due to the simultaneous extraction of epoxy-rich droplets embedded within the PEI-rich domains. As seen in Figure 7, these droplets constitute most of the volume of the PEI-rich domain. Therefore, the extracted mass cannot be directly used to quantitatively determine the degree of cocontinuity. Nevertheless, the reproducible mass removal across samples with widely different sizes strongly suggests that the solvent fully penetrated the sample volume. This is because, with different surface-to-volume ratios, a solvent action limited only to the exposed surface would be expected to produce different extracted fractions. Additionally, the formation of a self-supporting porous epoxy-rich scaffold after extraction shown in Figure S6 further suggests the presence of an interconnected morphology.

The coconnectivity was then further assessed by confocal microscopy. The same sample surface was imaged using laser and also visible light, which shines through the translucent matrix and is partially reflected back by the GNP flakes. This allows for a visualization of where the PEI-rich phase is located beneath the surface. As shown in Figure 12 for the IPN20 GNP 1 wt % sample, side-by-side comparisons of the laser (a) and visible light (b) images reveal shiny paths (GNP-filled domains) bridging the PEI-rich regions beneath the surface. These paths, highlighted with dashed lines, appear across almost every PEI-rich domain in the imaged area, indicating a high level of connectivity. Therefore, although connectivity is inherently a three-dimensional property, the combination of multiple 2D SEM observations, selective extraction experiments, and light/laser confocal imaging provided consistent evidence that the PEI-rich phase remained sufficiently continuous and that the observed reduction in electrical performance was not caused by a lack of phase continuity. This strengthens the claim that the epoxy-rich droplets are the main cause of nanoparticles’ connectivity disruption.

**Figure 12:**
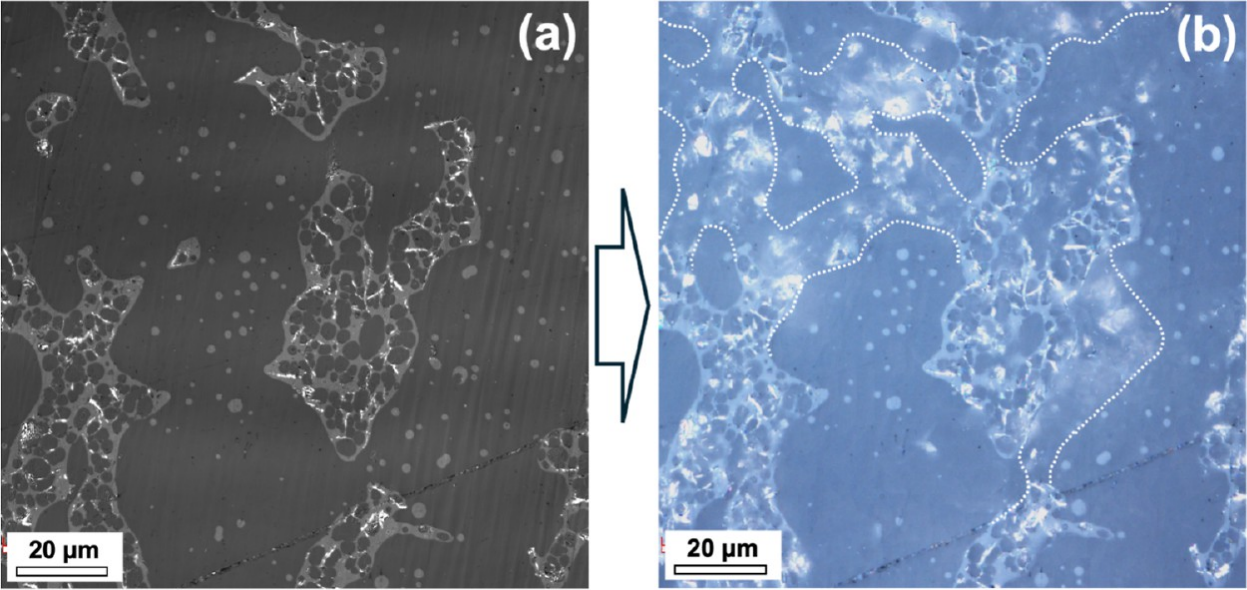
Confocal micrographs of the IPN20 GNP 1.0% sample, taken using green laser (a) and visible light (b) as the light source. Dashed lines in (b) highlight the interconnecting paths that lie beneath the surface, invisible in (a).

Finally, attempts were made to prevent or reduce the formation of epoxy-rich droplets and improve the conductivity of GNP-filled samples. Because the system’s composition remained fixed, only the curing temperature and PEI content could be adjusted to explore its phase diagram and potentially avoid secondary phase separation. However, as shown in Figure S7, changing the curing temperature did not have a significant enough impact to alter the dielectric character of the sample, and the PEI content of 20 PHR was already optimal. This suggests that epoxy-rich droplet formation is an inherent feature of the system used.

### 3.5 Impact Strength

#### 3.5.1 Thermoplastic Effect on Impact Strength of Unfilled Semi-IPNs

One of the most important advantages of semi-IPN systems over conventional thermosets is that the presence of a thermoplastic-rich second phase can provide the material with increased toughness, without harming their high thermal stability.

Figure 13 displays the impact strength of EP-PEI semi-IPNs as a function of PEI content, measured by the Izod method. Clearly, there is an optimal PEI content that maximizes toughness: at 15 PHR the impact strength increases by approximately 60%, while the other PEI contents tested actually caused a decrease in impact strength compared to the neat epoxy. A similar result was observed by Liu et al., in which small amounts of PEI (5%) reduced impact strength by 20%, while adding 13% of PEI led to a 34% increase.^61^ To make sense of these results, it is important to note that the presence of inclusion-like particles in a continuous matrix can be both beneficial and detrimental to the material’s toughness. On the one hand, the inherent difference in the linear coefficients of thermal expansion can create thermal stresses at the matrix–inclusion interface, resulting in stress concentrations and interfacial microcracks. On the other hand, a crack-pinning mechanism can also take place, where inclusions behave as obstacles to crack propagation and force the crack to deflect, dissipating more energy.^35^ Specifically for EP-PEI semi-IPNs, Ma and colleagues noticed that dispersed morphology can undergo two distinct fracture behaviors. In the first, the interaction of a propagating crack with the PEI particle leads to debonding, where the thermoplastic inclusion remains attached to one of the faces and leaves its “socket” on the other; in the second, the crack promotes plastic deformation and subsequent rupture of the PEI particle without debonding, leading to higher energy absorption.^36^ Therefore, the overall effect on toughness depends on the properties and morphologies of each phase, as well as the interaction between them.

**Figure 13:**
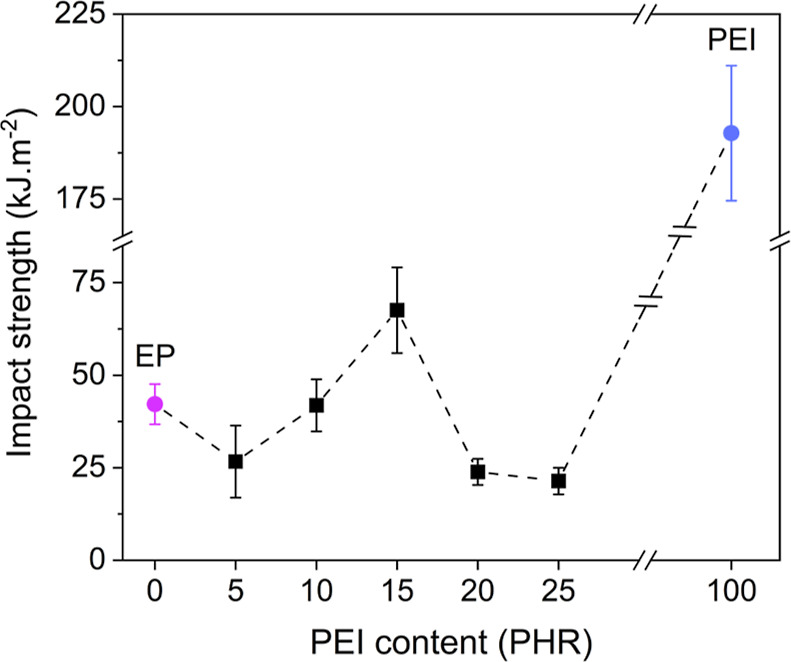
Impact strength of epoxy and EP-PEI semi-IPNs with varying PEI contents, compared to the neat epoxy resin (EP) and neat PEI.

Figure 14 displays SEM micrographs of fractured surfaces of epoxy and semi-IPNs with varying PEI contents. The epoxy micrograph (a) displays a smooth surface with the presence of river markings, typical of a brittle fracture.^25^ At the lowest PEI content tested, 5 PHR, the micrograph (b) shows that small and sparse PEI particles are present. The overall aspect of the surface remains mostly smooth, denoting a brittle fracture, but with the appearance of more river markings. These might be caused by the nonuniformity in the stress distribution ahead of the crack tip due to the PEI particles,^36^ which possibly acted as stress concentration points that caused the observed decrease in toughness. The micrographs of samples with 10 and 15 PHR of PEI displayed in (c) and (d) clearly show more river markings and, most importantly, larger PEI particles, which undergo one of the two fracture mechanisms described earlier: solid circles were drawn to highlight instances where debonding occurred, while dashed circles indicate particle rupture. These mechanisms are schematically illustrated in Figure 15. Clearly, at 10 PHR, the most common mechanism is debonding, while at 15 PHR it is particle rupture. This drastic change in fracture mechanisms explains why the impact strength at 15 PHR was significantly improved.

**Figure 14:**
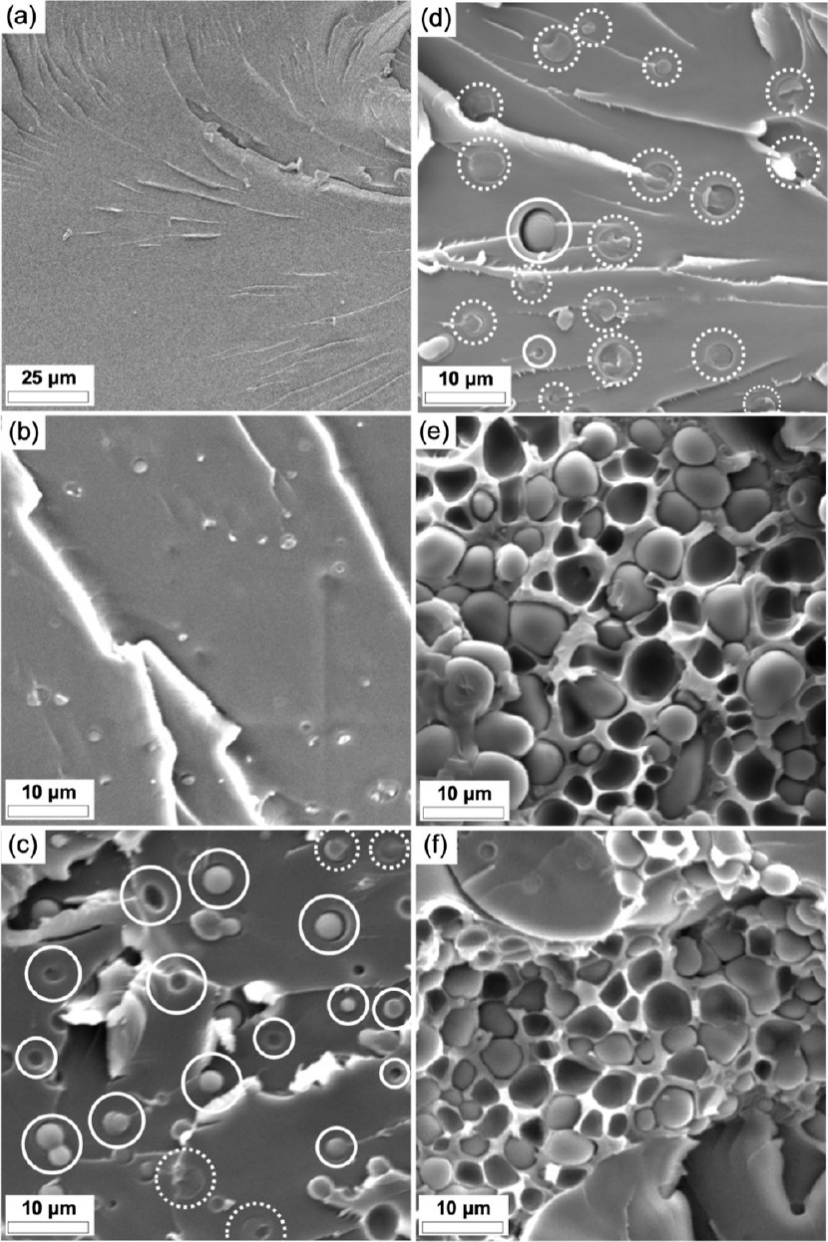
SEM micrographs of fractured surfaces of epoxy (a) and semi-IPN samples with varying PEI contents: 5 PHR (b), 10 PHR (c), 15 PHR (d), 20 PHR (e) and 25 PHR (f). Solid and dashed circles in (c) and (d) highlight examples of PEI particles that debonded and ruptured, respectively.

**Figure 15:**
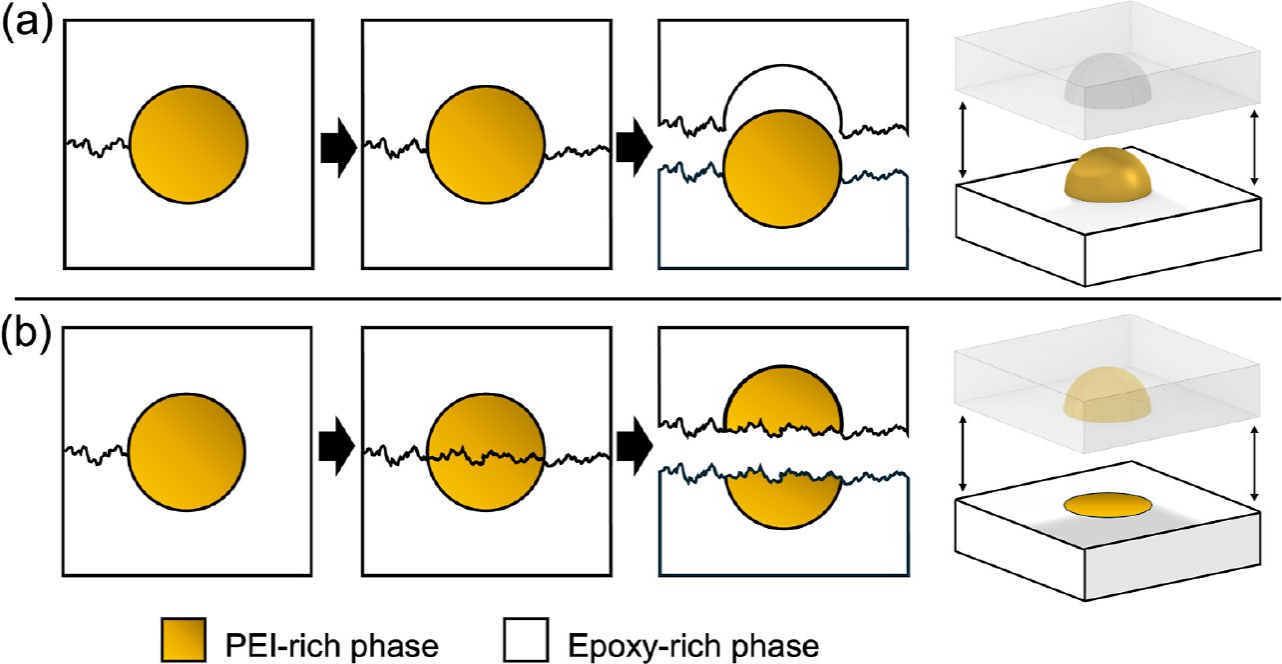
Fracture behavior of inclusion-like thermoplastic particles dispersed in epoxy resin: (a) debonding from the matrix and (b) rupture of thermoplastic.

At 20 PHR, the cocontinuous morphology is achieved, and the familiar presence of continuous PEI-rich regions filled with epoxy-rich droplets is seen in Figure 14e. With this morphology, the rigid epoxy-rich droplets remain undisturbed by crack propagation and always debond from their PEI-rich matrix host. Thus, the large volume occupied by these droplets cannot contribute significantly to resisting crack propagation, leading to a dramatic decrease in impact strength seen in Figure 13. At 25 PHR, the material has a phase-inverted morphology with more epoxy-rich droplets present, resulting in a slightly lower impact strength. The majority of published studies on epoxy-PEI semi-IPNs found that toughness increases significantly when a cocontinuous or phase-inverted morphology is achieved,^35,36,61–63^ rather than the detrimental effect seen here. However, toughness was assessed in those papers by single-edge notch beam (SENB) tests instead of impact tests, which do not involve crack initiation and have a significantly slower deformation rate. In addition, most of these studies employed epoxies with higher functionalities that are known to be more brittle and thus would benefit more from PEI addition. Those that used bifunctional epoxies have cured them with amine-based hardeners^36,63,64^ rather than an anhydride-based one, altering the chemistry and interface interactions between the phases. Thus, direct comparisons with the literature are limited. However, in the present epoxy-PEI system and under the high deformation rates of the Izod impact conditions employed here, the optimal impact strength is achieved when the morphology is well-dispersed and approaches (but does not reach) cocontinuity.

#### 3.5.2 Impact Strength of GNP-Filled Nanocomposites

Although GNPs can enhance toughness and impact resistance in epoxy-based nanocomposites by crack deflection mechanisms,^65–67^ they can also restrict plastic deformation of the matrix and act as stress concentration points, leading instead to a decrease in toughness, especially when agglomerated.^68^ As shown in Figure 16a, the latter effect seems to prevail: the addition of GNPs drastically decreased the impact strength in epoxy-based nanocomposites. Figure 17 shows that the introduction of GNPs dramatically altered the surface appearance of the fractured surfaces. Neat epoxy exhibits a smooth, brittle fracture surface with some river markings, typical of a brittle failure. In contrast, the addition of 1 wt % GNPs results in a rougher fracture surface with visible ditches caused by the pull-out of GNP flakes—an effect that becomes more pronounced at 5 wt % loading. This decrease in toughness can be attributed to the poor interfacial adhesion between the epoxy and nonfunctionalized GNPs, which promotes crack initiation and propagation at the filler–matrix interface.

**Figure 16:**
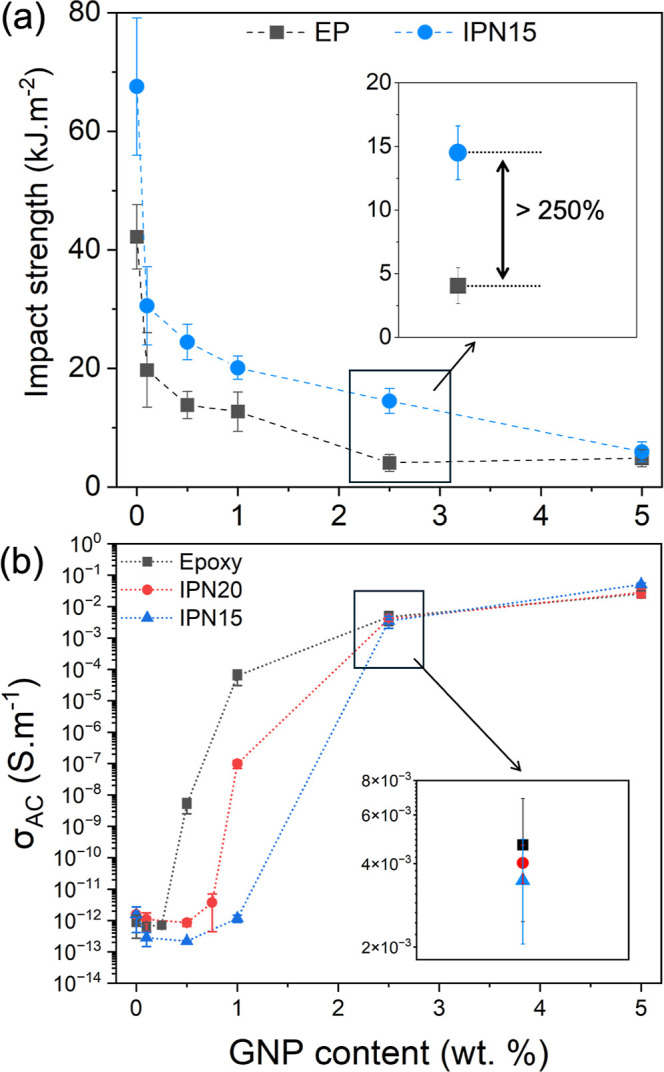
Impact strength (a) and electrical conductivity (b) as a function of GNP content for epoxy (EP) and semi-IPN matrices.

**Figure 17:**
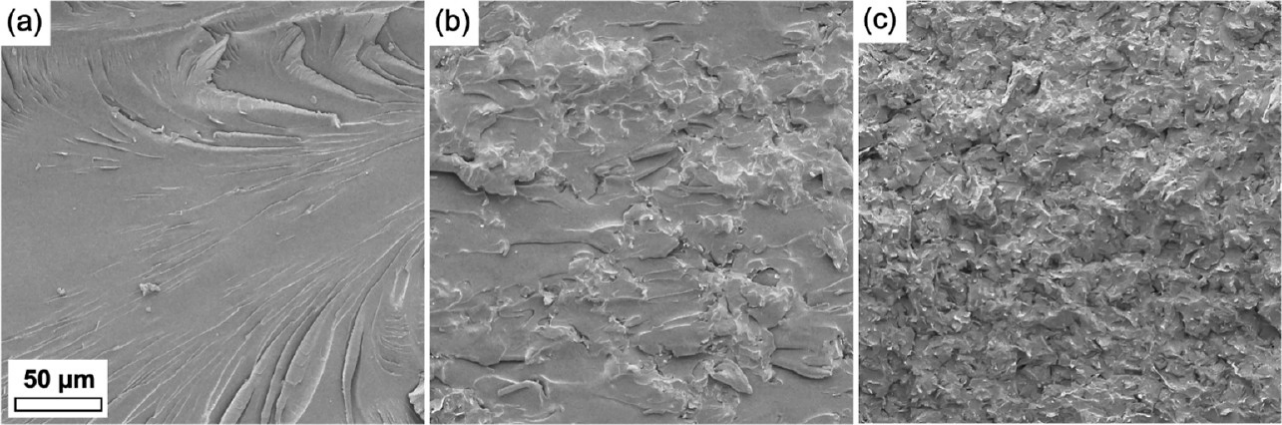
SEM micrographs from fractured surfaces of neat epoxy (a), EP 1 wt % (b) and EP 5 wt % (c) samples.

Given that 15 PHR of PEI yielded the highest toughness in the IPN system, this composition (IPN15) was selected to evaluate whether the interpenetrating polymer network could mitigate the negative effect caused by the GNPs. As shown in Figure 16a, although the addition of GNPs still led to a decrease in toughness, the extent of this reduction was substantially lower. At 2.5 wt % GNP loading, the impact strength was over 250% higher than that of the corresponding epoxy-based nanocomposite. Thus, not only did the IPN15 system increase toughness by 60% compared to neat epoxy, but it also mitigated the adverse effects of GNP incorporation.

Encouraged by this result, a new electrical percolation curve was obtained using the IPN15 matrix (Figure 16b). The spaced-out, dispersed PEI-rich domains in this formulation further disrupted GNP connectivity compared to the IPN20 system, pushing the percolation threshold to a higher weight fraction. Nevertheless, the electrical conductivity at 2.5 wt % GNPs remained nearly identical to that of the epoxy–GNP system. This finding shows that the IPN15 GNP 2.5 wt % formulation of IPN15 GNP offers a valuable combination of properties, maintaining electrical conductivity while delivering more than three times greater energy dissipation during fracture.

Figure 18 presents SEM micrographs of fractured surfaces for the IPN15 system at various GNP loadings. As discussed previously, crack propagation in this formulation occurs through both the PEI-rich dispersed phase and the epoxy-rich matrix (Figure 15b), thus increasing energy dissipation. Up to 1.0 wt % loading, the PEI-rich phase gradually becomes populated by the GNP flakes without substantial morphological changes. At 2.5 wt %, the number of PEI-rich spherical domains is significantly reduced as GNP flakes occupy a much larger volume and start to form interconnected networks, correlated with the sharp increase in conductivity. The trend continues at 5 wt %, with PEI-rich spheres no longer visible, point at which the fracture strength matches that of the unmodified epoxy-GNP nanocomposites.

**Figure 18:**
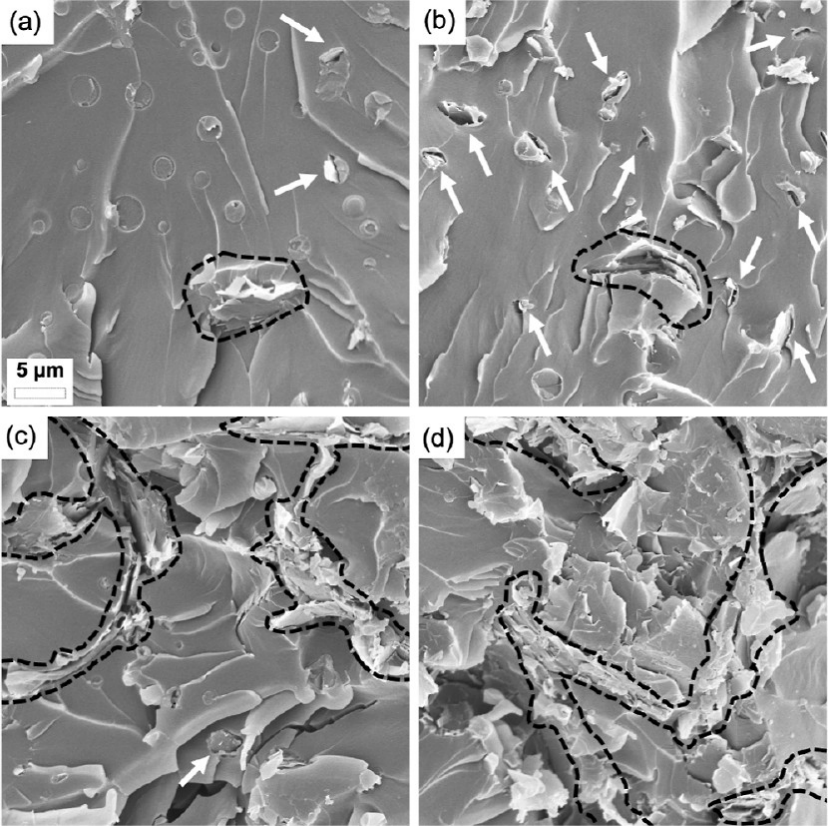
SEM micrographs from fractured surfaces of IPN15-based nanocomposites reinforced with 0.1, 1, 2.5, and 5 wt % of GNP, (a–d), respectively. White arrows point to GNP flakes locked inside PEI-rich dispersed phase, while dark dashed lines highlight larger GNP–GNP structures.

## 4 Conclusions

This study explored the use of GNP, CNT, and CB as conductive fillers in epoxy–PEI semi-interpenetrating polymer networks, with the aim of evaluating the potential of this matrix to simultaneously enhance electrical conductivity and impact strength for multifunctional aerospace applications. Key findings include:

- Cocontinuous morphologies were achieved at 20–21 PHR PEI, while dispersed and phase-inverted morphologies occurred at lower and higher PEI contents, respectively.
- All three nanofillers were successfully localized within the cocontinuous PEI-rich phase, as predicted by thermodynamic calculations and confirmed by microscopy and DMA analyses.
- Despite the combination of selective localization and phase cocontinuity, the expected double-percolation effect was not achieved. Electrical and morphological analyses indicate that secondary phase separation within the PEI-rich phase generated epoxy-rich droplets that disrupted conductive pathway formation and increased the effective separation between conductive domains. The claim that epoxy-rich droplets are the primary obstacles to conductive network formation is supported by the strong correlation between droplet presence and nanoparticle connectivity loss across different nanofillers, demonstrating that mesoscopic morphology plays a critical role in the design of electrically conductive multiphase nanocomposites.
- In contrast, a clear enhancement in toughness was observed in terms of impact strength. The IPN15 matrix exhibited the highest toughness, with a 60% increase in impact strength relative to neat epoxy. When reinforced with 2.5 wt % GNP, this formulation preserved the high electrical conductivity of the epoxy-based nanocomposite while achieving more than a 3-fold increase in energy dissipation during fracture.

In summary, the results demonstrate that phase cocontinuity and selective nanofiller localization, although important prerequisites for double percolation, are not sufficient to guarantee its occurrence in immiscible polymer systems. At the same time, the epoxy–PEI semi-IPN approach proved effective for improving toughness while maintaining useful levels of electrical conductivity. The results highlight the critical influence of mesoscopic morphology on the electrical behavior of multiphase nanocomposites and provide important insights into both the opportunities and limitations of epoxy–PEI semi-IPNs for multifunctional material design.

## Supplementary Material



## Notes

FTIR spectra of semi-IPN components, GNP and CNT characterization, optical microscopy monitoring of the reaction-induced phase separation (RIPS) process, solvent extraction method, effect of temperature and PEI content on GNP-filled semi-IPNs, quantitative analysis of the droplets’ morphology (PDF)
