# Supplementary material for: Morphological Constraints on Double Percolation and Toughening in Nanocarbon-Filled Epoxy–Poly(ether imide) IPN Nanocomposites
Source: ACS Omega. 2026 Jul 26;11(31):46322–39. doi: 10.1021/acsomega.6c03532 (PMC13470705; doi:10.1021/acsomega.6c03532)
Supplement: Supplementary file 1 [file ao6c03532_si_001.pdf]

SUPPORTING INFORMATION

Morphological constraints on double percolation and toughening in nanocarbon-filled epoxy–polyetherimide IPN nanocomposites

Matheus Mendes de Oliveira†, Rogerio Ramos de Sousa Jr†, Daria Strugova‡, Nicole R. Demarquette‡, Ragnar Larsson¶, Roland Kádár¶, Per Hallander§, and Danilo Justino Carastan†

† Center for Engineering, Modeling and Applied Social Sciences, Universidade Federal do ABC, Santo André, 09210-170, Brazil
‡ Mechanical Engineering Department, École de Technologie Supérieure, Montréal, H3C 1K3, Canada
¶ Industrial and Materials Sciences, Chalmers University of Technology, Göteborg, 412 96, Sweden
§ SAAB AB, Linköping, 581 88, Sweden

1. FTIR spectra of semi-IPN components and residual solvent assessment

FTIR spectra were taken for all the materials that make up the matrices used in this work, i.e., the thermoset components (DGEBA resin and MTHPA hardener) and the thermoplastic (PEI), and are displayed in **Figure S1**. The goal is to confirm that all relevant functional groups are present and that the starting materials are free of contaminants or compounds that were not disclosed in the product's data sheet.

The PEI spectrum displays bands at 1720 and 1780 cm^{-1} due to the carbonyl symmetric and asymmetric stretching, coming from the phthalimide groups [1], [2]. Bands at 2970 cm^{-1} are caused by CH stretching from propylidene moieties. The ether groups contribute to the 1070 cm^{-1} (stretching of aryl-ether-aryl bonds) [3], which also appear as vibrations at 1268, 1234, 1072, and 1014 cm^{-1} [1]. The spectrum as a whole matches what was reported previously for commercially pure PEI, with no foreign bands [1].

The DGEBA epoxy resin displays the characteristic oxirane absorption band at 914 cm^{-1}. The aromatic carbons absorb at 830, 1032 (together with aliphatic ether), 1182, 1502 and 1602 cm^{-1}, and the ether bonds at 1230-1250 cm^{-1}. The high-frequency bands at 2850-2960 cm^{-1} are due to the alkane bonds. The scattering pattern matches perfectly what has been reported for DGEBA-only epoxy resins, and, therefore, there are no aliphatic epoxies mixed in, unlike many commercial products [4].

Finally, for the MTHPA hardener, the absorption bands at 1857 and 1775 cm^{-1} are due to the stretching of CO groups and the 1109 cm^{-1} band comes from stretching of C-O-C bonds, both from the anhydride group (O=C-O-C=O). Bands at 1952 and 2932 cm^{-1} are associated with the stretch vibrations of C-H bonds [5], [6]. The cyclic anhydride group present in MTHPA also contributes to the absorption peaks at 1860 and 1760 cm^{-1} [7].

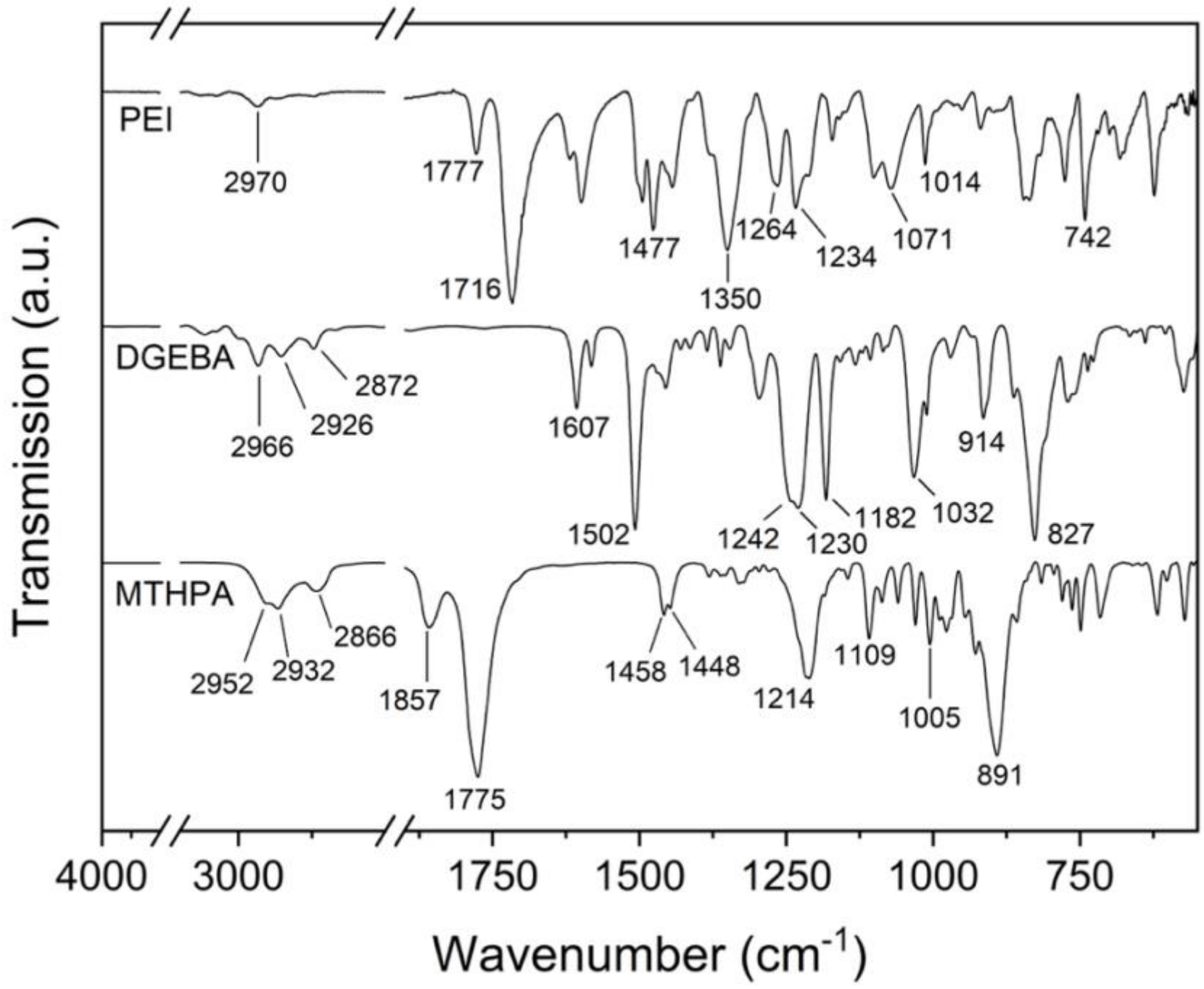

Figure S1. FTIR spectra of the three polymeric components used to produce the matrices. Regions without features were cut-out for clarity.

Dichloromethane (DCM) was used during the semi-IPN preparation process to dissolve PEI and enable homogeneous mixing with the DGEBA epoxy prepolymer. FTIR analysis was performed to evaluate whether the two solvent-removal steps (hotplate evaporation followed by overnight vacuum drying) were sufficient to eliminate residual DCM. This method has been successfully used to detect and quantify residual solvent [8].

Figure S2 presents the FTIR spectra of the neat IPN20 matrix and its GNP-filled nanocomposites containing 0.5 and 2.5 wt.% GNP, taken after addition of the curing agent and prior to curing. The spectra are compared with that of neat DCM. DCM exhibits characteristic absorption bands at 731 and 703 cm^{-1}, assigned to C–Cl stretching vibrations, as well as a band at 1264 cm^{-1} associated with CH_2 wagging vibrations from $-CH_2Cl$ [2]. Given the high sensitivity of FTIR, the absence of these characteristic DCM bands in the nanocomposites indicates that no significant amount of solvent remained after sample preparation.

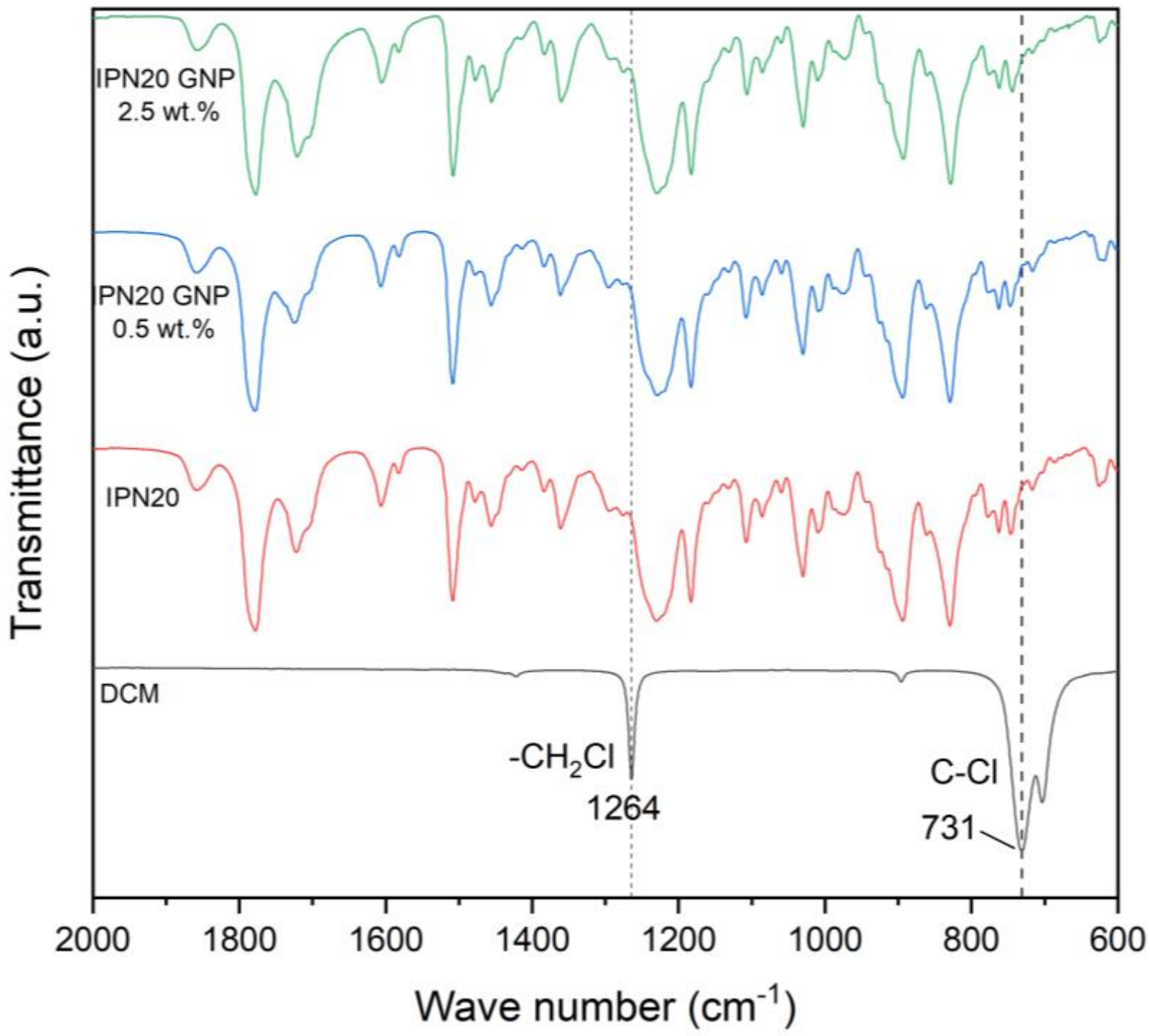

Figure S2. FTIR spectra of neat DCM, neat IPN20 matrix, and GNP-filled IPN20 nanocomposites containing 0.5 and 2.5 wt.% GNP, collected after addition of the curing agent and prior to curing.

2. GNP and CNT characterization

Multiple SEM images of GNPs were taken in order to measure about 450 individual flakes and build the lateral size distribution shown in **Figure S3** (a), following the recommended method described by the National Physical Laboratory (UK) [9]. Peaking at 1.68 µm, the lateral dimensions are within the expected wide range for GNPs (100 nm to 100 µm [10]). The distribution width is also adequate for GNPs, since they are known to contain graphene-related materials with a wide range of sizes (from few-layer graphene to nanostructured graphite [11], [12], [13]). AFM scans were used to measure the average thickness of the flakes. Around 80 representative flakes were measured, with the Gaussian distribution peaking at an average thickness of 5.64 nm. It must be noted that the height is dependent on the substrate, and, for SiO2/silicon wafers, heights of 1 nm have been reported for monolayer graphene (instead of the expected 0.355 nm of a single interatomic distance) [14]. Taking that into account and the graphitic interlayer distance, the flakes are between 14 and 16 layers thick on average.

Raman spectroscopy shown in **Figure S3** (c) give us more insight about the GNP's inner structure. The G peak at around 1582 cm^{-1} is associated with in-plane vibrations from sp^2-hybridized carbon atoms, while the D peak around 1350 cm^{-1} is associated with sp^3-hybridized carbon [15]. **Figure S3** (c) shows that I_DI_G ratios of representative flakes is small (< 0.2), which suggests a highly ordered carbon crystalline structure and thus high intrinsic conductivity. The shape of G' peak around 2720 cm^{-1} is especially sensitive to the number of graphene layers. Shape and position of the G' peak obtained clearly confirms that the GNP flakes are more than 5 layers thick (insert), as expected for this kind of nanomaterial and in agreement with the AFM results.

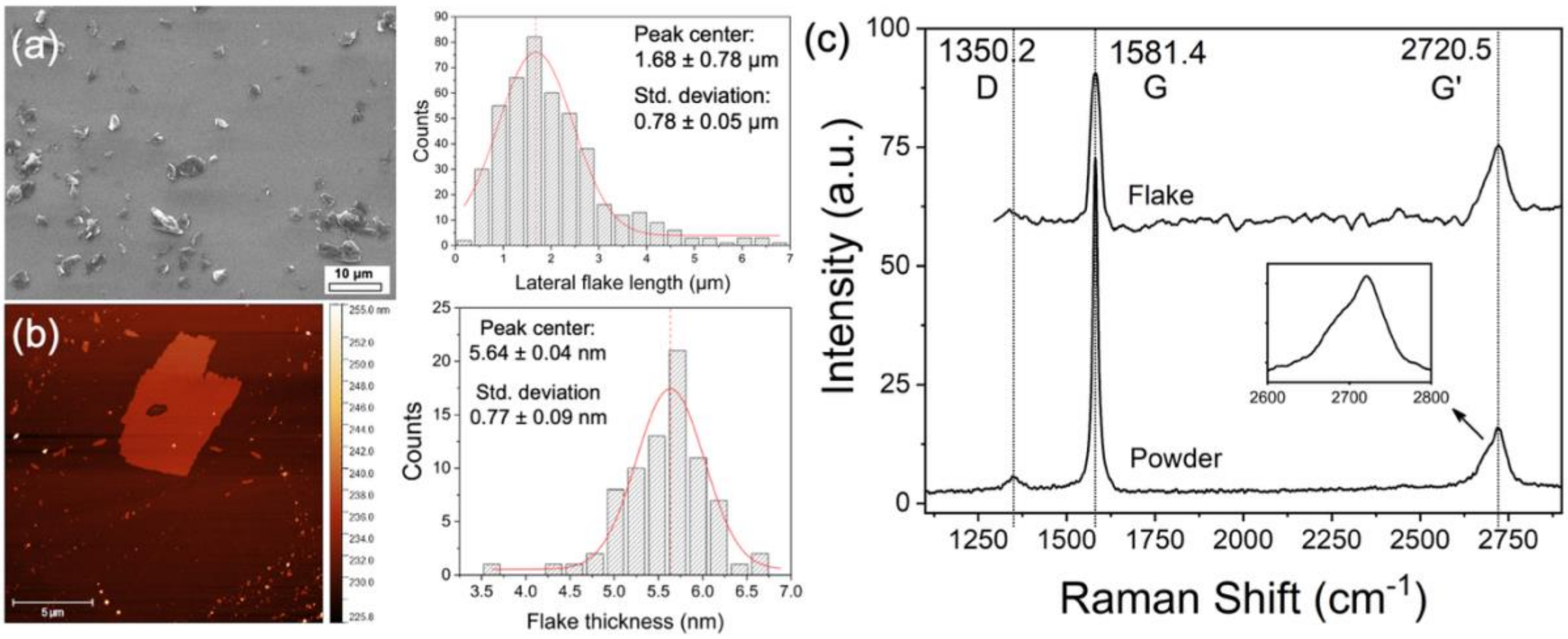

Figure S3. The top row (a) displays a representative SEM micrograph of GNP flakes on left, alongside the histogram of lateral sizes measured on the right. The bottom row (b) shows a representative AFM micrograph on the left and the respective histogram of measured heights. Both histograms were fitted with a Gaussian function to extract the peak center and the standard deviation. (c) Scattering Raman spectra of the as-received GNP and its relevant D, G and G' peaks. Bottom spectrum was taken from GNP powder, and the top spectrum from a representative individual GNP flake.

In order to obtain a diameter size distribution of the as-received CNTs, about 200 individual nanotubes were measured using field-emission scanning electron microscopy (FE-SEM). **Figure S4** shows that the Gaussian distribution peaked at 12.9 nm, which is close to the mean diameter informed by the manufacturer (9.5 nm) in the data sheet.

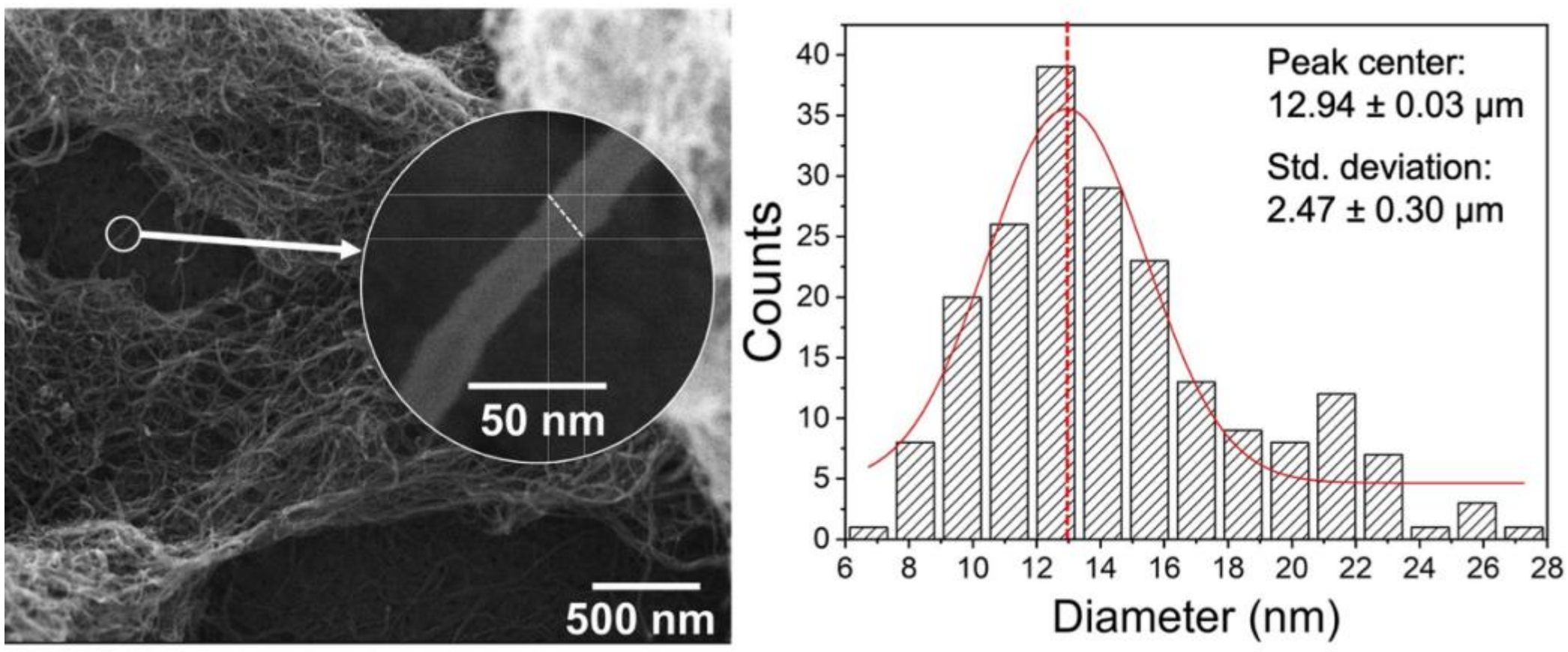

Figure S4. SEM micrograph of a CNT bundle from which the diameter of individual CNTs was measured to build the histogram on the right-hand side.

3. Reaction-induced phase separation (RIPS) process

Figure S5 shows the time evolution of RIPS of epoxy-PEI semi-IPNs at three different weight contents of PEI that are representative of each final morphology, all taken at the constant curing temperature of 110 ºC. In all three samples, the initial mixture is completely homogeneous. After approximately 5-6 min, features of a spinodal decomposition process appears uniformly across the imaged area. The initial co-continuity evolves into a dispersed

morphology after coalescence and coarsening in the 14 and 25 PHR samples, while the 20 PHR sample retains its bi-continuous structure until gelation freezes the morphology. Spinodal decomposition was also observed for all other PEI contents tested.

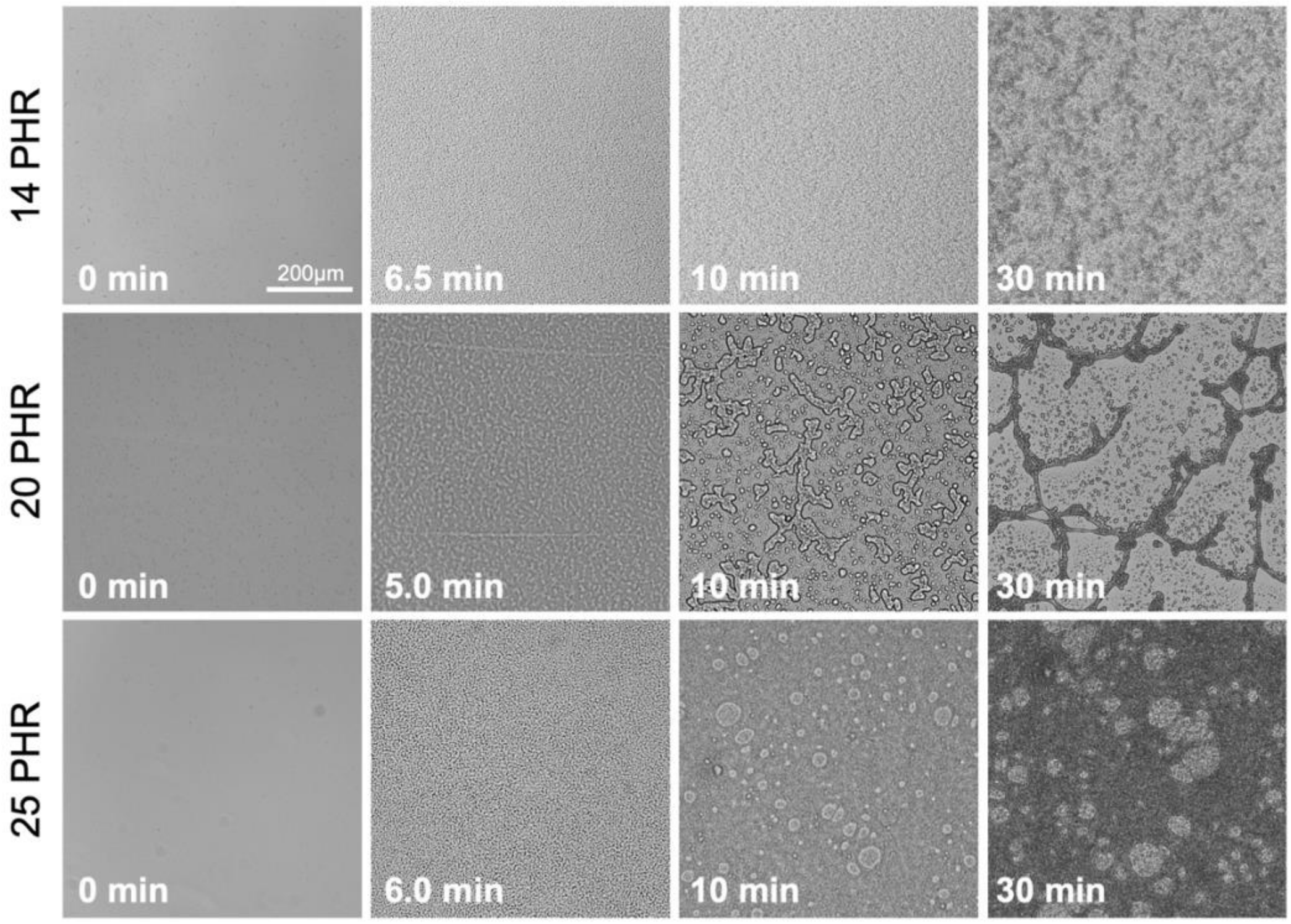

Figure S5. Morphology evolution as a function of time, for PEI contents of 14, 20 and 25 PHR content. Each condition results in a different final morphology: dispersed, co-continuous and phase-inverted, respectively, but all three go through a spinodal decomposition phase-separation process.

4. Solvent extraction

The solvent extraction method is based on placing a piece of material into a solvent that selectively removes only one of the phases. If the part remaining after extraction is still self-supporting and its mass corresponds to the nominal content of that phase, then the material can be considered co-continuous. If the extracted mass is less than expected, the degree of co-continuity can be calculated as the ratio between the extracted mass of that phase and its total content [16]. In order to assess the degree of co-continuity, the tests were restricted to the IPN20 without fillers and GNP-filled IPN20 samples with contents below percolation. IPN20 samples filled with 0.10, 0.25 and 0.50 wt.% of GNP were cut into three pieces of different sizes (small, medium and large), weighed and placed in DCM for 12 hours to extract the PEI-rich phase. In **Figure S6** (a) and (b) it is possible to see that the pieces, initially completely black, became white and/or grayish while the DCM solution darkened due to the removal of GNPs together with the PEI-rich phase. The total extracted mass for all pieces of all three specimens shown in **Table S1** is fairly consistent, averaging at about 40 %, despite the nominal PEI content being only ~9.7 %. As discussed in the manuscript, this higher loss is due to the simultaneous extraction of epoxy-rich droplets embedded within the PEI-rich domains.

IPN20 GNP 0.1 wt.%		IPN20 GNP 0.25 wt.%		IPN20 GNP 0.5 wt.%	
**Piece size**	**Mass Loss**	**Piece size**	**Mass Loss**	**Piece size**	**Mass Loss**
Small	40.0 %	Small	34.3 %	Small	41.2 %
Medium	39.3 %	Medium	40.7 %	Medium	38.3 %
Large	42.8 %	Large	40.8 %	Large	41.6 %
Average	40.7 %	Average	38.6 %	Average	40.4 %

Table S1. Mass loss after solvent extraction with DCM for GNP-filled IPN20 samples.

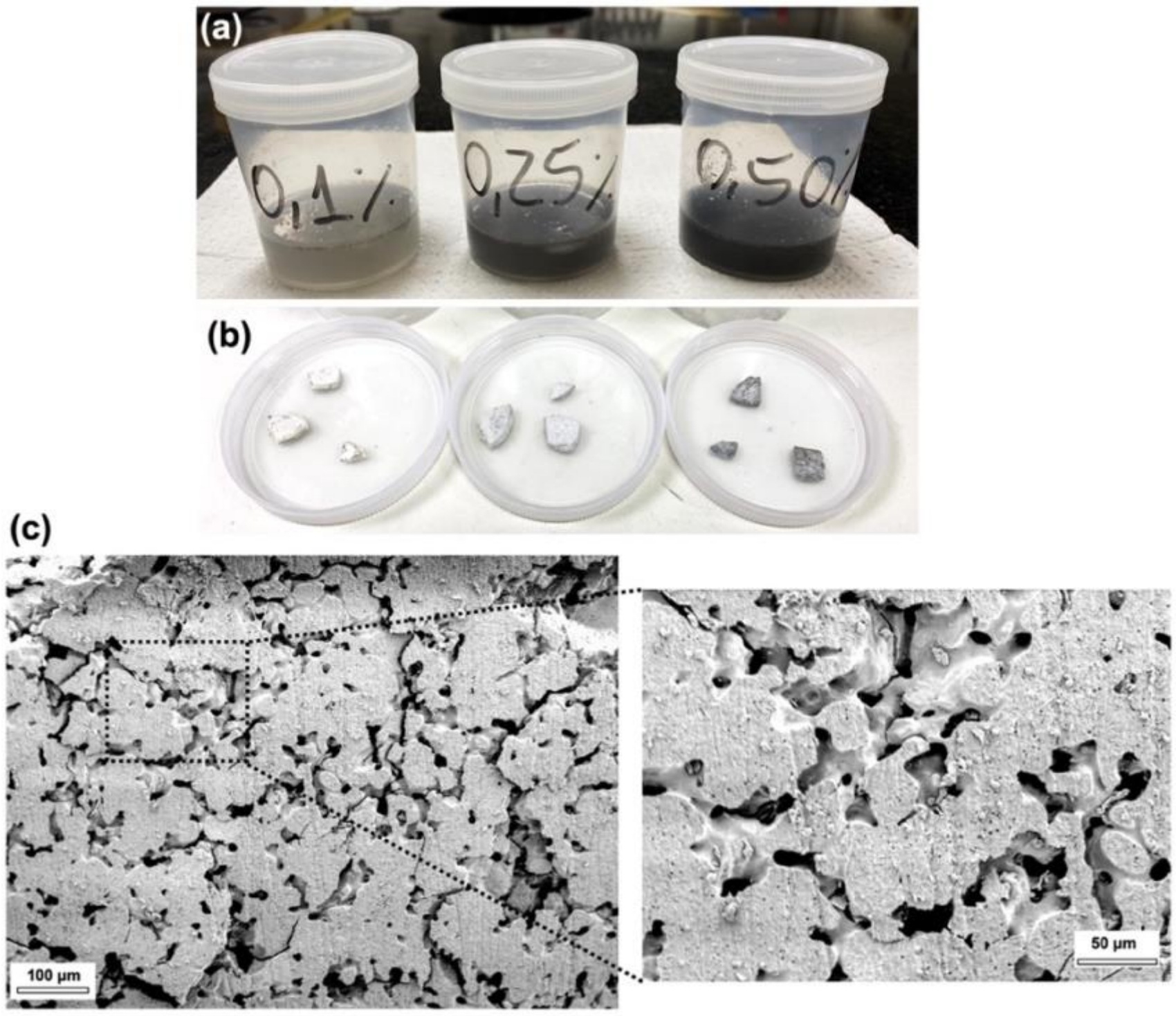

Figure S6. SEM micrograph of a IPN20 specimen after the solvent extraction with DCM. The remaining material is composed of the epoxy-rich phase, while the voids left are the removed PEI-rich phase.

5. Effect of temperature and PEI on GNP-filled semi-IPNs

Attempts were made to reduce or eliminate the epoxy-rich droplets to see if this would improve the conductivity of the GNP-filled samples. Without altering the composition of the samples, temperature and PEI content were the only factors that could be adjusted to explore the material's phase diagram and potentially prevent secondary phase separation. First, samples with the same IPN20 matrix were prepared and cured at 90, 110 and 130 ºC, without nanofillers. The RIPS was monitored through OM and the resulting micrographs are shown in **Figure S7** (a)-(d). There is a noticeable reduction in the number of epoxy-rich droplets at lower

curing temperatures (80 and 90 ºC), although they are still very numerous. Additionally, the morphology of the sample cured at 80 ºC is closer to phase-inverted rather than co-continuous. Next, samples with 0.75 wt.% were prepared and cured at the other three temperatures (90, 110 and 130 ºC). This GNP content was chosen because, since this is in the vicinity of percolation, the resulting conductivity changes are maximized and would be easier to detect. **Figure S7** (e) shows that while the different temperatures resulted in slightly different conductivities, these changes are rather small and the samples' conductivity remain orders of magnitude lower than its epoxy-matrix counterpart. Therefore, although the temperature did affect the secondary phase separation, it was not enough to significantly suppress the epoxy-rich droplets formation and improve electrical conductivity.

Then, the effect of PEI content was tested by preparing GNP-filled semi-IPN samples at 16, 18, 21 and 23 PHR, while keeping the original curing temperature of 110 ºC. The weight content of GNP was increased to 1.0 wt.% to bring the material even closer to the percolation threshold**. Figure S7** (f) shows the effect that varying the PEI content had on the morphology and electrical conductivity. At 16 PHR, the morphology reveals dispersed, unconnected PEI-rich droplets, resulting in the material remaining highly insulating. Although it is a droplet-free morphology, that is only because there is not PEI enough to form continuous PEI-rich domains in the first place. At 18 PHR, the morphology is undergoing a transition from a dispersed to co-continuous structure, leading to an improvement in conductivity of two orders of magnitude, albeit still significantly lower than the conductivity of its epoxy-matrix counterpart. Increasing the PEI content to 20 PHR further improves conductivity by fully establishing the PEI-rich phase's co-continuity. However, for the higher concentrations of 21 and 23 PHR, there is a significant drop in conductivity, despite the morphology of these samples remaining co-continuous. Thus, although changing the PEI content led to changes in conductivity/morphology, the optimal PEI range of 18-20 PHR is still less conductive than the GNP-filled epoxy-matrix samples.

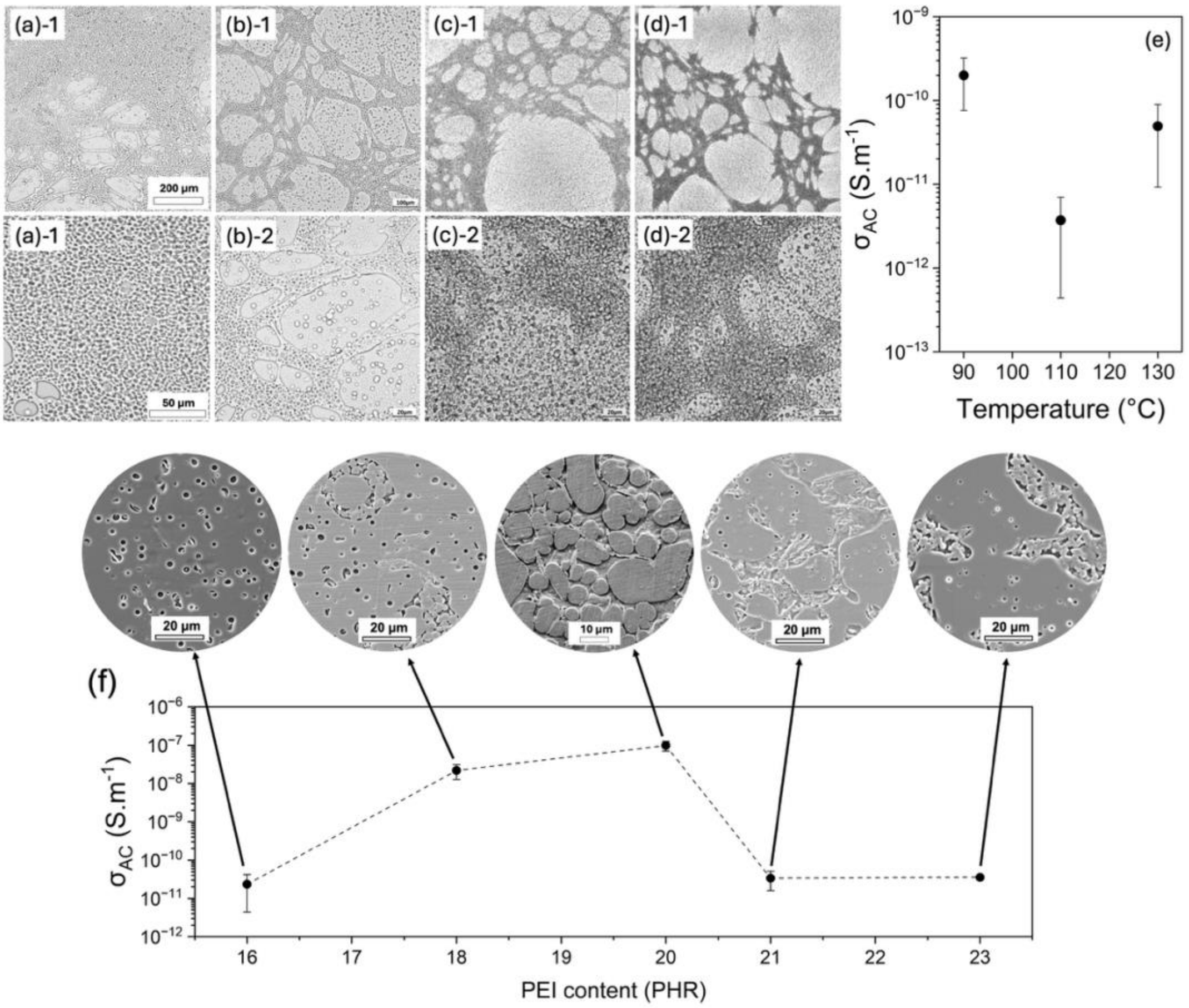

Figure S7. Optical micrographs of semi-IPNs containing 20 PHR of PEI, cured at varying temperatures: (a) 80 ºC, (b) 90 ºC, (c) 110 ºC and (d) 130 ºC. (e) displays the electrical conductivity of IPN20 filled with 0.75 wt.% of GNP as a function of curing temperature. (f) shows the effect of PEI content on the morphology (SEM micrographs on the top) and electrical conductivity of semi-IPNs filled with GNPs at 1.0 wt.%.

6. Quantitative analysis of the droplets' morphology

A quantitative analysis of the size and prevalence of epoxy-rich droplets was performed using the cross-sectional SEM images of the GNP- and CNT-filled IPN20 samples shown in **Figures S8 and S9**, respectively. For each condition, the diameters of several hundred droplets were measured using ImageJ software. Since the cross-sectional plane is unlikely to pass through the center of most droplets, the measured diameters generally underestimate their true size. However, the goal of this analysis was not to determine the absolute droplet dimensions, but rather to assess relative changes in droplet size with increasing nanofiller content. In addition, the total number of droplets within the imaged area was counted, allowing an estimation of the area fraction occupied by the epoxy-rich phase. The Delesse principle states that, for randomly distributed isotropic spheres, the area fraction measured on a 2D cross-section is equal to the corresponding 3D volume fraction [17].

The results, presented in Figure 11 in the manuscript, quantitatively demonstrate that in the GNP-filled semi-IPNs, both droplet size and prevalence decrease sharply around the electrical

percolation. In contrast, the CNT-filled systems exhibit no comparable reduction, with large numbers of droplets persisting throughout the entire concentration range investigated. These findings further support the conclusion that the epoxy-rich droplets are the primary factor governing the distinct percolation behavior observed in the CNT-filled semi-IPNs.

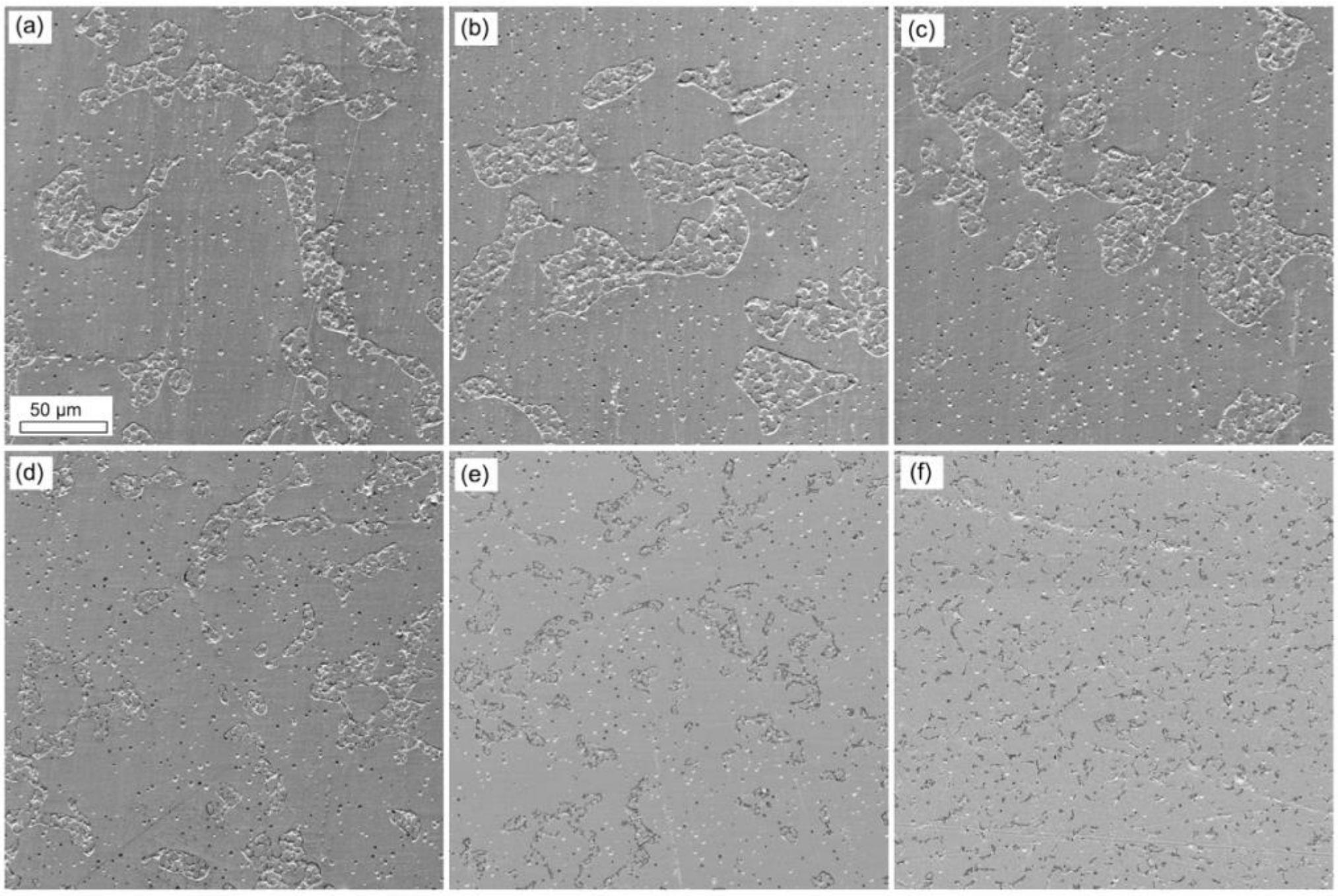

Figure S8. Cross-sectional SEM images of the GNP-filled IPN20 samples. Figures (a) through (f) show samples containing 0, 0.1, 0.5, 1.0, 2.5 and 5.0 wt.% of GNP, respectively.

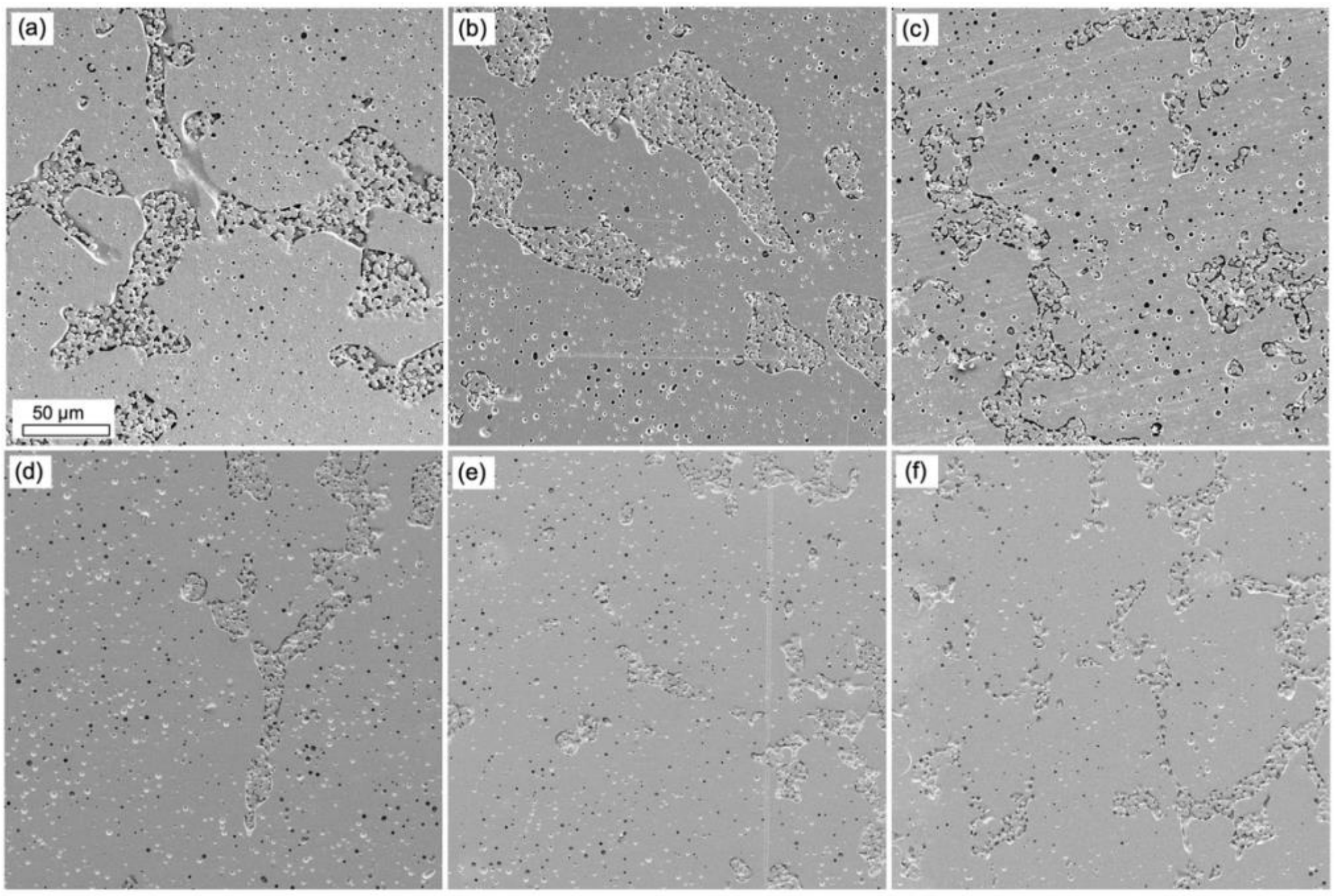

Figure S9. Cross-sectional SEM images of the CNT-filled IPN20 samples. Figures (a) through (f) show samples containing 0.05, 0.075, 0.10, 0.125, 0.150 and 0.20 wt.% of CNT, respectively.

References

[1] A. I. Romero, M. L. Parentis, A. C. Habert, and E. E. Gonzo, “Synthesis of polyetherimide/silica hybrid membranes by the sol–gel process: influence of the reaction conditions on the membrane properties,” *J. Mater. Sci.*, vol. 46, no. 13, pp. 4701–4709, Jul. 2011, doi: 10.1007/s10853-011-5380-4.

[2] G. Socrates, *Infrared and Raman Characteristic Group Frequencies: Tables and Charts*, 3rd ed. Chichester ; New York: Wiley, 2001.

[3] S. T. Amancio-Filho, J. Roeder, S. P. Nunes, J. F. Dos Santos, and F. Beckmann, “Thermal degradation of polyetherimide joined by friction riveting (FricRiveting). Part I: Influence of rotation speed,” *Polym. Degrad. Stab.*, vol. 93, no. 8, pp. 1529–1538, Aug. 2008, doi: 10.1016/j.polymdegradstab.2008.05.019.

[4] S. Meure, D.-Y. Wu, and S. A. Furman, “FTIR study of bonding between a thermoplastic healing agent and a mendable epoxy resin,” *Vib. Spectrosc.*, vol. 52, no. 1, pp. 10–15, Jan. 2010, doi: 10.1016/j.vibspec.2009.09.005.

[5] A. I. Barabanova *et al.*, “Cycloaliphatic epoxy resin cured with anhydride in the absence of catalyst,” *Colloid Polym. Sci.*, vol. 297, no. 3, pp. 409–416, Mar. 2019, doi: 10.1007/s00396-018-4430-8.

[6] J. Pu, C. Liu, S. Shi, and J. Yun, “Hydrogenation of MTHPA to MHHPA over Ni-based catalysts: Al_2O_3 coating, Ru incorporation and kinetics,” *RSC Adv.*, vol. 12, no. 53, pp. 34268–34281, 2022, doi: 10.1039/D2RA06738B.

[7] Daimay Lin-Vien, William G. Fateley, Norman B. Colthup, and Jeanette G. Grasselli, *The Handbook of Infrared and Raman Characteristic Frequencies of Organic Molecules*. Boston: Academic Press, 1991.

[8] M. M. De Oliveira, S. Forsberg, L. Selegård, and D. J. Carastan, “The Influence of Sonication Processing Conditions on Electrical and Mechanical Properties of Single and Hybrid Epoxy Nanocomposites Filled with Carbon Nanoparticles,” *Polymers*, vol. 13, no. 23, p. 4128, Nov. 2021, doi: 10.3390/polym13234128.

[9] A. J. Pollard, K. R. Paton, C. A. Clifford, and E. Legge, “Good practice guide No 145 - Characterization of the structure of graphene,” The National Physical Laboratory (NPL), Middlesex, UK, 2017.

[10] ISO, “ISO/TS 80004-13:2017 - Nanotechnologies — Vocabulary — Part 13: Graphene and related two- dimensional (2D) materials,” 2017.

[11] P. Cataldi, A. Athanassiou, and I. S. Bayer, “Graphene nanoplatelets-based advanced materials and recent progress in sustainable applications,” *Appl. Sci. Switz.*, vol. 8, no. 9, 2018, doi: 10.3390/app8091438.

[12] B. Z. Jang and A. Zhamu, “Processing of nanographene platelets (NGPs) and NGP nanocomposites: A review,” *J. Mater. Sci.*, vol. 43, no. 15, pp. 5092–5101, 2008, doi: 10.1007/s10853-008-2755-2.

[13] R. Sengupta, M. Bhattacharya, S. Bandyopadhyay, and A. K. Bhowmick, “A review on the mechanical and electrical properties of graphite and modified graphite reinforced polymer composites,” *Prog. Polym. Sci. Oxf.*, vol. 36, no. 5, pp. 638–670, 2011, doi: 10.1016/j.progpolymsci.2010.11.003.

[14] F. Bonaccorso, A. Lombardo, T. Hasan, Z. Sun, L. Colombo, and A. C. Ferrari, “Production and processing of graphene and 2d crystals,” *Mater. Today*, vol. 15, no. 12, pp. 564–589, Dec. 2012, doi: 10.1016/S1369-7021(13)70014-2.

[15] S. Reich and C. Thomsen, “Raman spectroscopy of graphite,” *Philos. Trans. R. Soc. Lond. Ser. Math. Phys. Eng. Sci.*, vol. 362, no. 1824, pp. 2271–2288, Nov. 2004, doi: 10.1098/rsta.2004.1454.

[16] P. Pötschke and D. R. Paul, “Formation of Co-continuous Structures in Melt-Mixed Immiscible Polymer Blends,” *J. Macromol. Sci. Part C Polym. Rev.*, vol. 43, no. 1, pp. 87–141, Jan. 2003, doi: 10.1081/mc-120018022.

[17] S. M. El-Soudani, “The fundamental equation of quantitative microstructural analysis,” *Metallography*, vol. 8, no. 4, pp. 297–327, Aug. 1975, doi: 10.1016/0026-0800(75)90032-4.
